# Supplementary material for: Deep learning to decode sites of RNA translation in normal and cancerous tissues
Source: Nat Commun. 2025 Feb 2;16:1275. doi: 10.1038/s41467-025-56543-0 (PMC11788427; doi:10.1038/s41467-025-56543-0)
Supplement: Supplementary file 1 — Supplementary Information [file 41467_2025_56543_MOESM1_ESM.pdf]

Supplementary Information and Figures

Deep learning to decode sites of RNA translation in normal and cancerous tissues

1. Supplementary Methods

1.1. Ribosome profiling processing

Cutadapt and STAR are applied for trimming adapters and mapping reads to the genome and transcriptome. Read lengths between 20 and 40 nucleotides are retained. Reads mapping against tRNA/rRNA/sm(o)RNA are filtered out. Extended Data Table 1 lists the total number of reads within the dataset at various steps. The data were selected in order to have variation with respect to applied treatments and mapped number of reads.

```
# trim files and perform fastqc
cutadapt -j 20 -m 20 -a $adapter ${dataset}.fastq -o "out/temp/${dataset}_trimmed.fq" > "
    ↪ out/temp/${dataset}_trimmed_report.txt"

# remove rRNA/tRNA/smRNA/smoRNA
STAR --genomeLoad NoSharedMemory --seedSearchStartLmaxOverLread .5 --genomeDir '../../
    ↪ genome/STAR/excl_RNA' --readFilesIn $trimmed --outFilterMultimapNmax 1000 --
    ↪ outFilterMismatchNmax 2 --outFileNamePrefix out/temp/ --runThreadN 20 --
    ↪ outReadsUnmapped Fastx
mv out/temp/Unmapped.out.mate1 $cleaned

# align to genome, output mapping to transcriptome as well
STAR --runThreadN 20 --genomeDir '../../genome/STAR' --genomeLoad NoSharedMemory --
    ↪ readFilesIn $cleaned --outFileNamePrefix out/ --outSAMtype BAM SortedByCoordinate
    ↪ --quantMode TranscriptomeSAM --outSAMattributes MD NH --outFilterMultimapNmax 10
    ↪ --outMultimapperOrder Random --outFilterMismatchNmax 2 --
    ↪ seedSearchStartLmaxOverLread 0.5 --alignEndsType EndToEnd --outWigType bedGraph
```

1.2. RiboTIE

1.2.1. Data processing

Data loading for RiboTIE is achieved by storing data in the hierarchical data format version 5 (*hdf5*). Using Python, the ribosome reads mapped to the transcriptome are stored by transcript. The generated *bam* files are parsed using Python and data is stored to the *hdf5* format. Data is aggregated by the total number of reads aligned by their 5' position for every read length and transcript position. Transcript matrices are loaded from the *hdf5* files by a PyTorch data loader object and used as inputs to the model.

1.2.2. Input embedding strategies

The transformer architecture takes full transcript regions as input and provides a prediction along each position of the input range. No sequence information is processed. No ORFs are identified as a pre-processing step. Transformer networks use vector representations of mapped reads at each nucleotide position as input tokens. As part of this research, different approaches were explored to create input vector representations from the mapped ribosome profiling data. For all instances, read counts are normalized for each transcript. This ensures the numerical stability of the inputs. Supplementary Figures 19 and Extended Data Figure 3 illustrate both strategies evaluated as part of this paper.

1.2.3. Model architecture

RiboTIE is built upon the architectural framework of TIS transformer[1], a transformer model used for predicting translation initiation sites using transcript sequence information. The transformer structure features multiple layers with multiple attention heads per layer. These adhere to an identical framework but feature unique trainable model parameters and approaches to calculate the input vectors. The outputs of the transformer module are sent to a set of fully connected layers to obtain a binary output at each input position. Notwithstanding the size of the dataset and overall high computational requirements of transformer architectures, model optimization **from scratch** is possible on a single RTX 3090 and converges after ca. 10 hours due to the relative shallowness of the transformer architecture as compared to many language-learning transformers. Model optimization using a pre-trained network converges <1h.

Algorithm 1 RiboTIE network architecture. Given are the different layers, their respective dimensions as defined by their hyperparameter names, the dimensions for RiboTIE (Table 4), and the resulting total weights. The bias term applied in each node is included and marked with italics.

RiboTIE | **211,964**
  Ribosome Read Count | **21,546**
    Linear | $1 \times \text{dim}$ | $1 \times 42 + \mathit{42}$ | **84**
    Linear | $\text{dim} \times \text{dim} * 6$ | $42 \times 252 + \mathit{252}$ | **10,836**
    Linear | $\text{dim} * 6 \times \text{dim}$ | $252 \times 42 + \mathit{42}$ | **10,626**
  Ribosome Read Count Embedding | $1 \times \text{dim}$ | 1×42 | **42**
  Ribosome Read Length Embedding | read lengths $\times$ dim | 21×42 | **882**
  Positional Embedding | fixed positional embeddings | **0**
  Performer | **185,712**
    Layer ($\times$ depth | 6) | **30,952**
      Layer norm | $\text{dim} \times 2$ | $42 \times 2 + \mathit{2}$ | **86**
      Attention head ($\times$ n_head | 6) | **2,064**
        $\mathbf{W_Q}$ | dim $\times$ dim_head | $42 \times 16 + \mathit{16}$ | **688**
        $\mathbf{W_K}$ | dim $\times$ dim_head | $42 \times 16 + \mathit{16}$ | **688**
        $\mathbf{W_V}$ | dim $\times$ dim_head | $42 \times 16 + \mathit{16}$ | **688**
      $\mathbf{W_o}$ | dim_head $*$ n_head $\times$ dim | $96 \times 42 + \mathit{42}$ | **4,074**
      Layer norm | $\text{dim} \times 2$ | $42 \times 2 + \mathit{2}$ | **86**
      Linear | $\text{dim} \times \text{dim} * 4$ | $42 \times 168 + \mathit{168}$ | **7,224**
      Linear | $\text{dim} * 4 \times \text{dim}$ | $168 \times 42 + \mathit{42}$ | **7,098**
  Linear | $\text{dim} \times \text{dim} * 2$ | $42 \times 84 + \mathit{84}$ | **3,612**
  Linear | $\text{dim} \times 2$ | $84 \times 2 + \mathit{2}$ | **170**

1.2.4. Attention

Custom attention strategies can be performed by the attention heads independent of the number of weights utilized to calculate the **Q**, **K**, **V** matrices. In this model, full attention is calculated through the Fast Attention Via Positive Orthogonal Random Features (FAVOR+) algorithm [2]. These allow full attention, where all inputs along the transcript are included by the attention head. In contrast, local attention restricts the attention matrix to only neighboring positions. Local attention is implemented by dividing the attention matrix in smaller blocks on which full attention is calculated. Three blocks around the evaluated input are calculated. These local attention heads do not apply the FAVOR+ algorithm and use rotary positional embeddings [3]. The block size of the local attention heads is referred to under the 'attention scheme' columns of Supplementary Table 4.

Plastid

```
reformat_transcripts --annotation_files genome/Homo_sapiens.GRCh38.107.gff3 --
    ↪ annotation_format GFF3 --output_format GTF2 genome/Homo_sapiens.GRCh38.107.gtf2
metagene generate genome/plastid/ --landmark cds_start --annotation_files genome/
    ↪ Homo_sapiens.GRCh38.107.gtf2
psite genome/plastid/_rois.txt ribo/${dataset}/out/plastid/ --min_length 20 --max_length
    ↪ 41 --require_upstream --count_files ribo/${dataset}/out/genome/${dataset}_aligned.
    ↪ bam
```

RiboWaltz

```
library(riboWaltz)

metadata <- read.table('ribo/metadata.txt', header = FALSE, sep = "", dec = ".")
annotation_db <- create_annotation('genome/Homo_sapiens.GRCh38.107.gtf')
for (i in metadata$V1){
    reads_list <- bamtolist(bamfolder=sprintf("ribo/%s/out/", i), annotation=annotation_db
        ↪ )
    filtered_list <- length_filter(data=reads_list, length_filter_mode="custom", length_
        ↪ range=20:40)
    psite_offset <- psite(filtered_list)
    dir.create(sprintf("ribo/%s/out/ribowaltz", i))
    write.table(psite_offset, sprintf("ribo/%s/out/ribowaltz/riboWaltz_offsets.csv", i),
        ↪ sep="\t")
}
```

1.2.5. Benchmark

Code snippets used to run various tools. ORFquant was run without use of any flags except those selecting the input and output files and is thus not listed.

PRICE

```
gedi -e IndexGenome -s genome/Homo_sapiens.GRCh38.dna.primary_assembly.fa -a genome/
    ↪ Homo_sapiens.GRCh38.107.gtf -f genome/price -nobowtie -nostar -nokallisto
gedi -e Price -reads ribo/${dataset}/out/genome/${dataset}_aligned.bam -genomic
    ↪ Homo_sapiens.GRCh38.107 -prefix ribo/${dataset}/out/price/ -progress -plot
```

Rp-Bp

```
prepare-rpbp-genome ../scripts/benchmark/rpbp_full.yml --star-options "--
    ↪ genomeSAindexNbases␣10" --mem 10G --num-cpus 4 --logging-level INFO --log-file
    ↪ genome/rpbp/rpbp-genome.log --write-unfiltered
run-all-rpbp-instances ribo/${dataset}/out/rpbp/rpbp.yml --num-cpus 30 --logging-level
    ↪ INFO --mem 50G
```

Ribo-TISH

```
ribotish quality -b ribo/${dataset}/out/genome/${dataset}_aligned.bam -g genome/
    ↪ Homo_sapiens.GRCh38.107.gtf -f ribo/${dataset}/out/ribotish/quality.pdf -r ribo/${
    ↪ dataset}/out/ribotish/offset.txt -o ribo/${dataset}/out/ribotish/quality.txt -l
    ↪ 20,41
ribotish predict -b ribo/${dataset}/out/genome/${dataset}_aligned.bam -g genome/
    ↪ Homo_sapiens.GRCh38.107.gtf -f genome/Homo_sapiens.GRCh38.dna.primary_assembly.fa
    ↪ -o ribo/${dataset}/out/ribotish/orfs.txt --ribopara ribo/${dataset}/out/ribotish/
    ↪ offset.txt
```

ribotricer

```
ribotricer prepare-orfs --gtf genome/Homo_sapiens.GRCh38.107.gtf --fasta genome/
    ↪ Homo_sapiens.GRCh38.dna.primary_assembly.fa --prefix genome/ribotricer/ribo
ribotricer detect-orfs --bam ribo/${dataset}/out/genome/${dataset}_aligned.bam --
    ↪ ribotricer_index genome/ribotricer/ribo_candidate_orfs.tsv --prefix ribo/${dataset
    ↪ }/out/ribotricer/
```

2. Supplementary Tables

Supplementary Table 1: **Various tools designed to detect expressed coding sequences using ribosome profiling data.** Methods with * require RNA-seq data. The list is non-exhaustive.

**Method**	**Author**	**Calling A/P-site**	**Calling ORF**	**Year**	**Last update**	**Language**
Ribotricer	[4]	Yes	Yes	2019	1.3.3 (2023)	Python
Ribodeblur	[5]	Yes	No	2018	2018	Python
RiboWaltz	[6]	Yes	No	2018	1.2.0 (2021)	R
RibORF	[7]	Yes	Yes	2018	2.0 (2022)	Perl
RiboCode	[8]	Yes	Yes	2018	1.2.15 (2022)	Python
RiboWave	[9]	Yes	Yes	2018	2018	Python
Scikit-ribo*	[10]	No	Yes	2018	2018	Python
Rp-Bp	[11]	Yes	Yes	2017	3.0.1 (2023)	Python
RiboTISH	[12]	Yes	Yes	2017	0.2.7 (2021)	Python
Plastid	[13]	Yes	No	2016	0.6.1 (2022)	Python
PRICE (Gedi)	[14]	No	Yes	2016	1.0.5 (2022)	Python
SPECtre	[15]	No	Yes	2016	1.0.0 (2018)	R/Python
riboHMM*	[16]	No	Yes	2016	2016	Python
RiboProfiling	[17]	Yes	Yes	2016	1.28.0 (2022)	R
RiboTaper*	[18]	No	Yes	2015	1.3 (2016)	R
ORF-RATER	[19]	No	Yes	2015	2018	Python2.7
PROTEOFORMER	[20]	Plastid	Yes	2015	2.0 (2022)	Python2.7/Perl

Supplementary Table 2: **A/P-offsets determined by various tools evaluated during this study.** The listed tools are Plastid (P), RiboWaltz (W), RiboCode (C), and RiboTISH (T)

	**SRR1802129**					**SRR2433794**					**SRR2732970**					**SRR2733100**				
	**%**	**P**	**W**	**C**	**T**	**%**	**P**	**W**	**C**	**T**	**%**	**P**	**W**	**C**	**T**	**%**	**P**	**W**	**C**	**T**
**20**	0.2	13	7	-	-	0.1	13	13	-	-	1.3	2	12	-	-	0.8	2	12	-	-
**21**	0.2	13	9	5	17	0.2	13	13	-	-	1.6	3	12	-	12	1.0	3	12	12	12
**22**	0.2	13	9	-	-	0.4	5	12	-	-	1.7	12	12	-	-	1.0	3	12	-	-
**23**	0.2	13	7	-	7	0.7	6	12	-	12	2.2	12	12	-	-	1.2	5	12	-	-
**24**	0.3	13	8	-	8	1.0	7	12	-	-	2.2	12	12	-	12	1.4	5	12	-	12
**25**	0.4	13	9	-	12	2.4	9	12	12	12	2.3	12	12	-	-	1.5	12	12	-	-
**26**	1.7	50	10	-	-	5.2	9	12	-	12	2.5	12	12	-	-	1.8	12	12	-	-
**27**	9.6	12	12	-	11	8.7	10	12	-	12	3.1	12	12	12	12	2.5	12	12	12	12
**28**	36.3	12	12	12	12	19.7	12	12	-	12	4.8	12	12	12	12	4.3	12	12	-	12
**29**	32.0	12	13	-	12	37.4	12	12	12	12	14.5	12	12	12	12	13.3	12	12	12	12
**30**	9.0	23	15	-	13	21.8	12	12	12	12	30.0	12	12	12	12	27.7	12	12	12	12
**31**	1.7	13	14	-	-	2.1	13	13	-	-	21.6	12	12	12	12	25.0	12	12	-	12
**32**	1.4	13	16	-	-	0.3	50	11	-	-	8.3	12	13	12	12	12.5	13	13	-	-
**33**	2.5	13	17	-	-	0.1	13	12	-	15	2.5	13	13	-	-	4.0	5	13	-	-
**34**	2.5	13	19	-	-	0.0	13	14	-	-	0.8	12	13	-	-	1.0	5	13	-	-
**35**	1.4	13	18	-	-	0.0	13	13	-	-	0.3	13	13	-	-	0.4	13	14	-	-
**36**	0.1	13	20	-	-	0.0	13	15	-	-	0.2	12	12	-	-	0.2	13	12	-	-
**37**	0.1	13	21	-	-	0.0	13	12	-	-	0.1	50	12	-	-	0.2	13	14	-	-
**38**	0.1	13	22	-	-	0.0	13	12	-	-	0.1	13	12	-	-	0.1	13	12	-	-
**39**	0.1	13	23	-	-	0.0	13	12	-	-	0.0	50	12	-	-	0.1	13	14	-	-
**40**	0.0	13	24	-	-	0.0	13	12	-	-	0.0	13	12	-	-	0.0	13	13	-	-

	**SRR2954800**					**SRR8449577**					**SRR9113067**					**SRR11005875**				
	**%**	**P**	**W**	**C**	**T**	**%**	**P**	**W**	**C**	**T**	**%**	**P**	**W**	**C**	**T**	**%**	**P**	**W**	**C**	**T**
**20**	0.0	13	7	-	9	1.0	5	12	-	-	4.6	3	12	-	12	0.1	13	12	-	12
**21**	0.0	13	11	-	8	1.6	5	12	-	12	6.8	12	12	12	12	0.4	13	12	-	-
**22**	0.1	11	11	-	-	1.6	6	12	12	12	4.7	29	13	-	-	0.8	8	12	-	-
**23**	0.1	13	12	-	-	1.7	7	12	-	-	3.2	3	12	-	12	1.3	6	12	-	12
**24**	0.6	5	11	-	11	2.0	8	12	-	-	3.1	3	12	-	12	1.7	7	12	-	-
**25**	1.4	6	12	-	-	2.9	9	12	12	12	3.5	12	12	-	12	2.1	8	12	-	-
**26**	2.1	7	11	-	-	6.3	11	12	-	-	4.3	3	12	-	12	3.1	9	12	-	12
**27**	3.3	11	11	8	11	15.7	12	12	-	-	5.4	12	12	-	12	5.3	11	12	-	-
**28**	4.7	11	11	-	-	43.2	12	12	12	12	8.8	12	12	-	12	23.5	12	12	12	12
**29**	10.5	11	11	11	11	19.2	12	12	12	12	17.4	12	12	12	12	45.7	12	12	12	12
**30**	26.9	11	12	-	12	2.5	6	12	12	12	19.0	12	12	-	12	13.7	12	12	12	12
**31**	21.0	12	12	-	12	0.9	13	12	12	12	11.5	13	13	-	12	1.5	50	12	-	-
**32**	9.8	12	12	-	12	0.5	13	12	-	12	4.9	10	13	-	-	0.3	13	12	-	-
**33**	5.9	11	12	-	-	0.4	13	12	-	-	2.0	13	11	-	-	0.2	13	12	-	-
**34**	4.5	12	12	-	-	0.2	13	13	12	-	0.5	13	14	-	-	0.1	13	12	-	-
**35**	3.4	13	12	-	-	0.1	13	12	-	-	0.2	13	12	-	-	0.0	13	12	-	12
**36**	2.0	50	12	-	-	0.1	13	14	-	-	0.1	13	11	-	-	0.0	13	10	-	-
**37**	1.2	13	11	-	-	0.0	13	11	-	-	0.1	13	11	-	-	0.0	13	18	-	-
**38**	1.0	13	13	-	-	0.0	13	12	-	-	0.1	13	12	-	-	0.0	13	12	-	-
**39**	1.1	13	11	-	-	0.0	13	12	-	-	0.0	13	12	-	-	0.0	13	12	-	-
**40**	0.3	13	13	-	-	0.0	13	12	-	-	-	13	12	-	-	0.0	13	12	-	-

Supplementary Table 3: **RiboTIE performances for different input token strategies and datasets**. Scores are calculated on the test set after selection of the model with the minimum validation loss (See Extended Data Figure 3). For each dataset and strategy, the cross-entropy loss ($\times 10^3$), area under the receiver operating characteristic curve (ROC), and area under the precision-recall curve (PR) are given. Results indicate the relevance of read length information for the prediction of translation initiation sites using ribosome profiling data, especially for datasets featuring a higher read depth (see Extended Data Table 1). All strategies are evaluated using the same model architecture and training/validation data (Architecture 4, see Supplementary Table 4, Supplementary Figure 20), Strategy A generates input tokens utilizing read count information for every position of the transcript. Strategy A includes mappings generated by taking the 5' position of every read, and offsetting reads based on read length utilizing two different tools (Plastid, RiboWaltz). Strategy B includes information on both the positions and read lengths of the mapped reads.

		**SRR1802129**			**SRR2433794**			**SRR2732970**			**SRR2733100**		
	**Position**	**Loss**	**ROC**	**PR**	**Loss**	**ROC**	**PR**	**Loss**	**ROC**	**PR**	**Loss**	**ROC**	**PR**
**A**	5'	1.71	0.938	0.0211	1.48	0.965	0.0937	1.40	0.963	0.161	1.38	0.965	0.161
**A**	Plastid	1.74	0.935	0.0144	1.49	0.964	0.0825	1.39	0.965	0.156	1.41	0.964	0.145
**A**	RiboWaltz	1.7	0.941	0.0205	1.47	0.966	0.0908	1.4	0.964	0.154	1.4	0.964	0.151
**B**	5'	**1.69**	0.945	0.0217	**1.44**	0.968	0.104	**1.31**	0.969	0.211	**1.3**	0.97	0.217

		**SRR2954800**			**SRR8449577**			**SRR9113067**			**SRR11005875**		
	**Position**	**Loss**	**ROC**	**PR**	**Loss**	**ROC**	**PR**	**Loss**	**ROC**	**PR**	**Loss**	**ROC**	**PR**
**A**	5'	1.69	0.935	0.0394	1.55	0.955	0.0721	1.7	0.943	0.0178	1.48	0.967	0.0727
**A**	Plastid	1.72	0.931	0.0309	1.57	0.954	0.0579	1.7	0.942	0.0161	1.5	0.966	0.0721
**A**	RiboWaltz	1.71	0.932	0.0324	1.56	0.955	0.064	1.7	0.943	0.0171	1.48	0.967	0.0721
**B**	5'	**1.69**	0.937	0.04	**1.54**	0.956	0.0751	**1.7**	0.943	0.0189	**1.47**	0.967	0.0804

Supplementary Table 4: **Eight model architectures used for hyperparameter tuning selection.** For each set-up, a model is trained to detect translation initiation sites using ribosome profiling data. Hyperparameter selection is based on the minimum loss on the validation set. Hyperparameter tuning is performed on SRR2733100, featuring the highest read depth of all evaluated datasets. Supplementary Figure 20 displays the validation loss curves for each of the listed architectures.

	**Hidden**		**Attention head**		**Attention scheme**			
**ID**	**state dim.**	**Depth**	**Heads**	**Head dim.**	**Local**	**Full**	**Model parameters**	**Val. loss ($\times 10^{-3}$)**
1	24	5	6	12	4	2	81K	1.099
2	30	6	6	16	4	2	129K	1.105
3	30	8	6	24	4	2	215K	1.108
**4**	**42**	**6**	**6**	**16**	**4**	**2**	**211K**	**1.095**
5	42	8	6	24	4	2	339K	1.099
6	48	6	8	16	5	3	297K	1.096
7	48	8	8	24	5	3	484K	1.110
8	50	10	10	24	6	4	525K	1.102

Supplementary Table 5: **RiboTIE performances for different model optimization strategies and datasets**. Scores are calculated on the test set after selection of the model with the minimum validation loss (See Supplementary Figure 22, 23). For each dataset and strategy, the cross-entropy loss ($\times 10^3$), area under the receiver operating characteristic curve (ROC), and area under the precision-recall curve (PR) are given. Results show the gain by having a model trained on a large variety of ribosome-profiling datasets using a supervised learning objective (see Extended Data Table 1). All settings are evaluated using the same model architecture (Architecture 4, see Supplementary Table 4) and input token strategy (Strategy B, see Supplementary Table 3. The data is split in two folds (F1 and F2), with different parts of the transcriptome covered as training/validation/test data in each fold.

		**SRR1802129**			**SRR2433794**			**SRR2732970**			**SRR2733100**		
	**Pre-train**	**Loss**	**ROC**	**PR**	**Loss**	**ROC**	**PR**	**Loss**	**ROC**	**PR**	**Loss**	**ROC**	**PR**
	-	1.58	0.944	0.017	1.36	0.968	0.097	1.24	0.969	0.193	1.22	0.969	0.203
**F1**	Supervised	**1.56**	0.948	0.024	**1.32**	0.970	0.120	**1.18**	0.972	0.239	**1.18**	0.972	0.240
	Self-Supervised	1.57	0.946	0.020	1.36	0.966	0.110	1.20	0.970	0.227	1.20	0.969	0.226
	-	1.66	0.944	0.015	1.47	0.962	0.084	1.29	0.968	0.193	1.29	0.969	0.195
**F2**	Supervised	**1.63**	0.948	0.024	**1.42**	0.967	0.104	**1.25**	0.972	0.227	**1.24**	0.972	0.229
	Self-Supervised	1.65	0.945	0.021	1.44	0.965	0.098	1.26	0.969	0.214	1.28	0.969	0.214

		**SRR2954800**			**SRR8449577**			**SRR9113067**			**SRR11005875**		
	**Pre-train**	**Loss**	**ROC**	**PR**	**Loss**	**ROC**	**PR**	**Loss**	**ROC**	**PR**	**Loss**	**ROC**	**PR**
	-	1.60	0.935	0.033	1.43	0.959	0.079	1.59	0.946	0.013	1.37	0.969	0.075
**F1**	Supervised	**1.57**	0.939	0.045	**1.40**	0.962	0.096	**1.55**	0.951	0.024	**1.34**	0.972	0.094
	Self-Supervised	1.59	0.936	0.036	1.42	0.959	0.087	1.58	0.946	0.019	1.37	0.968	0.077
	-	1.70	0.932	0.025	1.52	0.956	0.071	1.68	0.941	0.013	1.45	0.966	0.076
**F2**	Supervised	**1.65**	0.939	0.044	**1.47**	0.960	0.092	**1.62**	0.949	0.027	**1.42**	0.970	0.090
	Self-Supervised	1.68	0.933	0.033	1.50	0.958	0.085	1.67	0.944	0.015	1.45	0.967	0.079

Supplementary Table 6: **Comparative performances of RiboTIE based on different subsets of the data**. The model predictions to detect translation initiation sites for each position on the trancriptome can be subsetted as a post-processing step. Using Ensembl translation initiation sites to derive a positive set, the area under the receiver operating characteristic curve (ROC) and area under the precision-recall curve (PR) are calculated. Given is the performance for all positions (total of ∼430M), with no conditions for what a valid ORF constitutes, positions that result in an ORF with a valid stop codon on the transcript (stop codon), an ORF length larger than 30 nucleotides (ORF length), and an ATG start codon (ATG start). Additionally, a subset has been selected using a minimum of 20 mapped reads (# Reads) on the transcript as a requirement. The performance and percentage of the total samples when using a combination of all listed conditions (Combined) is listed in the last set of columns. Note that the predictions of RiboTIE are those of the models pre-trained using a supervised learning strategy (see Supplementary Table 5), where the predictions of both models/folds are simply merged to cover the full transcriptome.

	**-**		**Stop codon**		**ORF length**		**# Reads**		**ATG start**		**Combined**		
**dataset**	**ROC**	**PR**	**ROC**	**PR**	**ROC**	**PR**	**ROC**	**PR**	**ROC**	**PR**	**%**	**ROC**	**PR**
SRR1802129	0.945	0.020	0.946	0.020	0.943	0.021	0.981	0.041	0.952	0.318	0.6	0.983	0.502
SRR2433794	0.965	0.101	0.966	0.103	0.964	0.103	0.983	0.137	0.966	0.419	1.0	0.981	0.516
SRR2732970	0.969	0.220	0.970	0.222	0.968	0.223	0.986	0.285	0.958	0.399	1.1	0.972	0.487
SRR2733100	0.969	0.215	0.970	0.217	0.968	0.218	0.986	0.279	0.957	0.399	1.1	0.971	0.488
SRR2954800	0.935	0.034	0.935	0.034	0.932	0.036	0.972	0.068	0.942	0.266	0.6	0.974	0.417
SRR8449577	0.958	0.083	0.959	0.084	0.957	0.085	0.983	0.128	0.961	0.382	0.8	0.982	0.514
SRR9113067	0.944	0.016	0.945	0.016	0.942	0.017	0.971	0.024	0.950	0.289	0.9	0.974	0.399
SRR11005875	0.967	0.077	0.968	0.078	0.966	0.080	0.985	0.106	0.969	0.432	1.0	0.984	0.534

3. Supplementary Figures

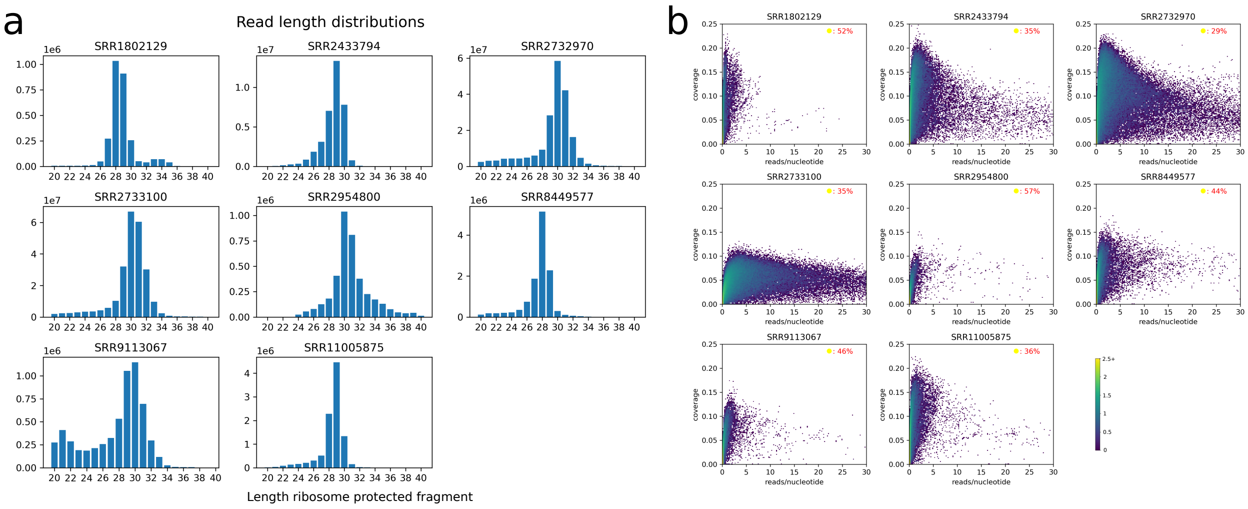

Supplementary Figure 1: **Benchmark dataset characteristics**. **a**, Read length distributions for the benchmark datasets for reads mapped to the genome. The most abundant read length is generally around 29 nucleotides. **b**, 2D histogram of coverage (y-axis) and number of reads per nucleotide (x-axis) for reads mapped to the transcriptome. The coverage is calculated based on the fraction of positions on the transcript that have at least one read mapped for reads mapped by their 5' position (i.e., every read covers one transcript position). The color map follows a logarithmic scale and is identical for all datasets. For each of the benchmark datasets, the percentage of transcripts in the transcriptome with no reads mapped is given (top-right corner)

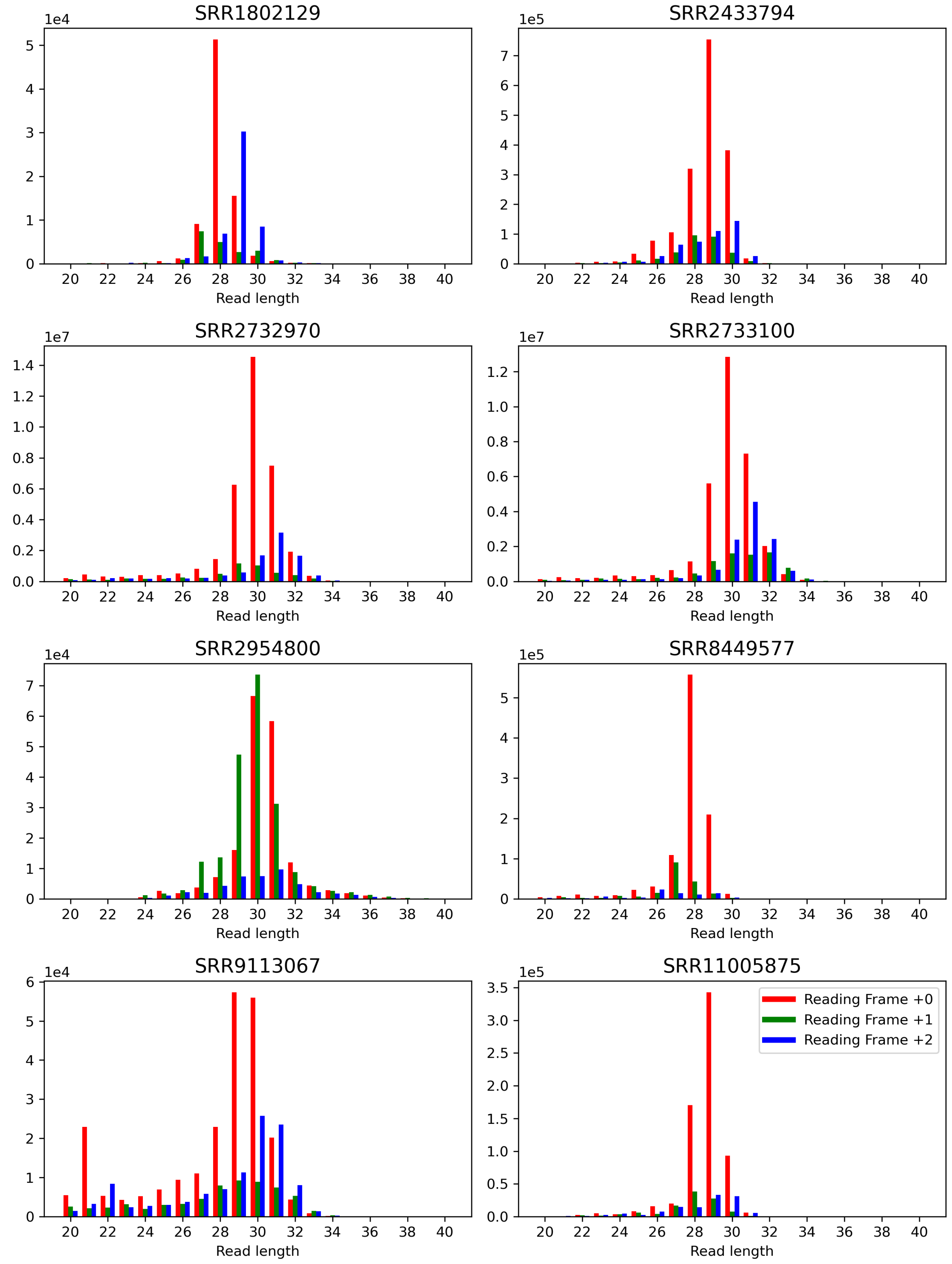

Supplementary Figure 2: **Read length counts binned by reading frame offset for all benchmark datasets.** Reads are mapped by their 5' positions. The figure highlights the skewed abundance of reads as influenced by the reading frame of the neighboring translation initiation site. Similar plots have been used to filter or offset the mapping position of reads in relation to their length. Read counts are taken by only evaluating translation initiation sites of coding sequences within the consensus coding sequence (CCDS) library. A window of 20 nucleotides upstream and 40 nucleotides downstream is taken to calculate the total read counts.

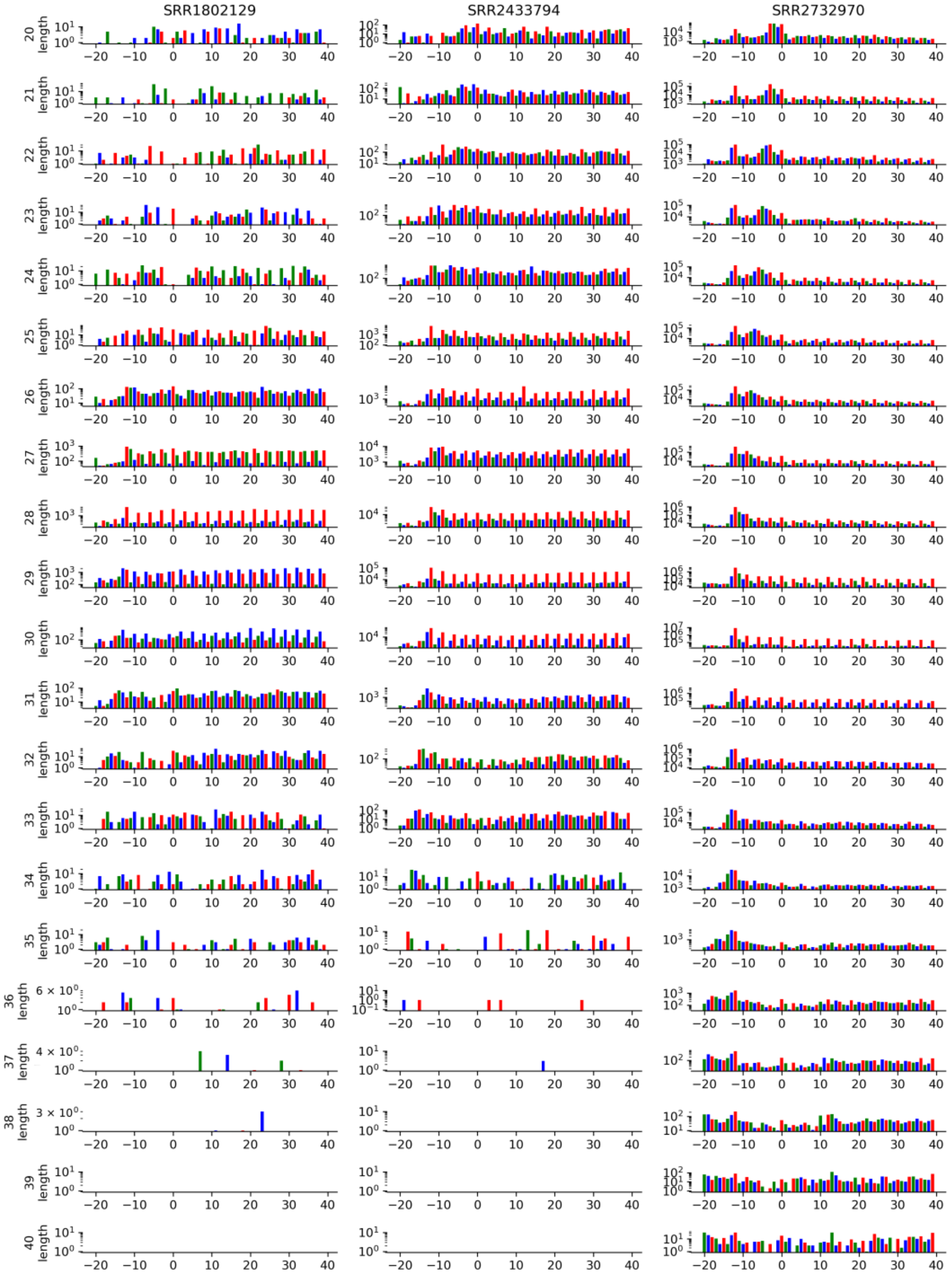

Supplementary Figure 3: **Counts of reads mapped by their 5' positions along translation initiation start sites.** The figure showcases unique patterns of read alignments per read length and experiment. Read counts are taken by only evaluating translation initiation sites of coding sequences within the consensus coding sequence (CCDS) library. A window of 20 nucleotides upstream and 40 nucleotides downstream is taken. A logarithmic scale and alternating color scheme is used to highlight the patterns emerging from the triplet periodicity along the translation initiation site and coding sequence. Included are experiments SRR1802129, SRR2433794, and SRR2732970. Accompanied by Supplementary Figure 4 and 5.

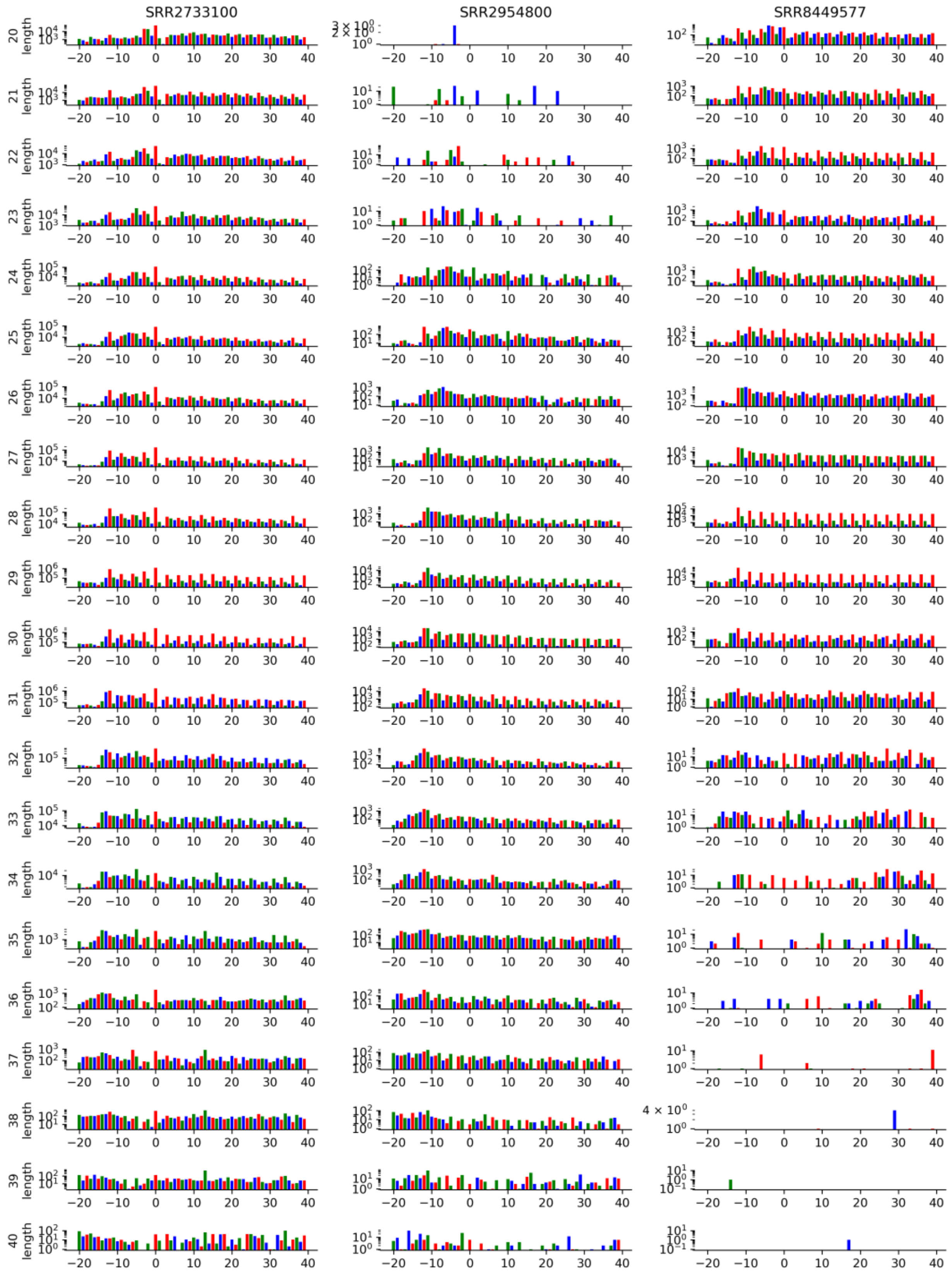

Supplementary Figure 4: **Counts of reads mapped by their 5' positions along translation initiation start sites.** The figure showcases unique patterns of read alignments per read length and experiment. Read counts are taken by only evaluating translation initiation sites of coding sequences within the consensus coding sequence (CCDS) library. A window of 20 nucleotides upstream and 40 nucleotides downstream is taken. A logarithmic scale and alternating color scheme is used to highlight the patterns emerging from the triplet periodicity along the translation initiation site and coding sequence. Included are experiments SRR2733100, SRR2954800, and SRR8449577. Accompanied by Supplementary Figure 3 and 5.

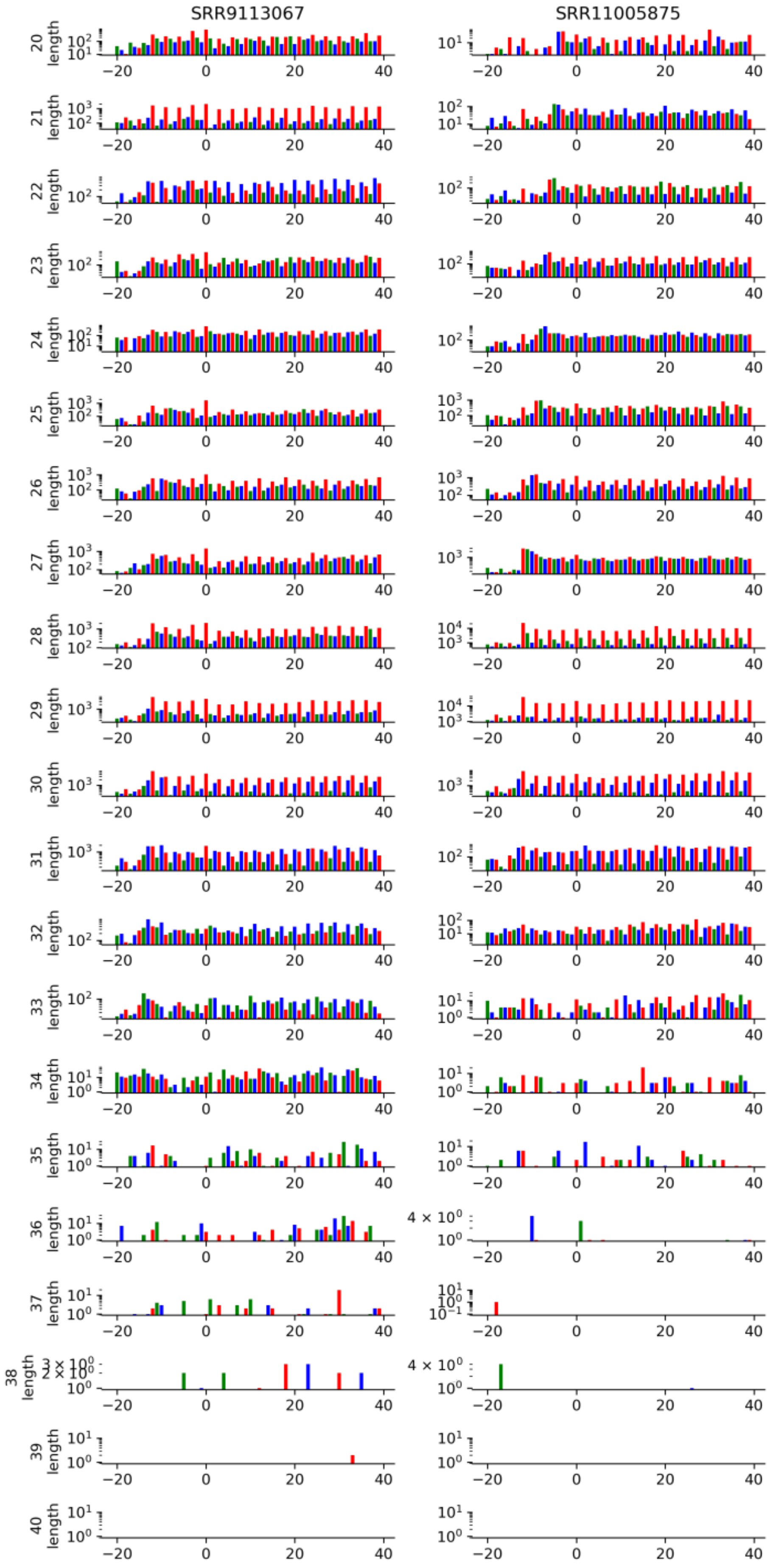

Supplementary Figure 5: **Counts of reads mapped by their 5' positions along translation initiation start sites.** The figure showcases unique patterns of read alignments per read length and experiment. Read counts are taken by only evaluating translation initiation sites of coding sequences within the consensus coding sequence (CCDS) library. A window of 20 nucleotides upstream and 40 nucleotides downstream is taken. A logarithmic scale and alternating color scheme is used to highlight the patterns emerging from the triplet periodicity along the translation initiation site and coding sequence. Included are experiments SRR9113067, SRR11005875. Accompanied by Supplementary Figure 3 and 4.

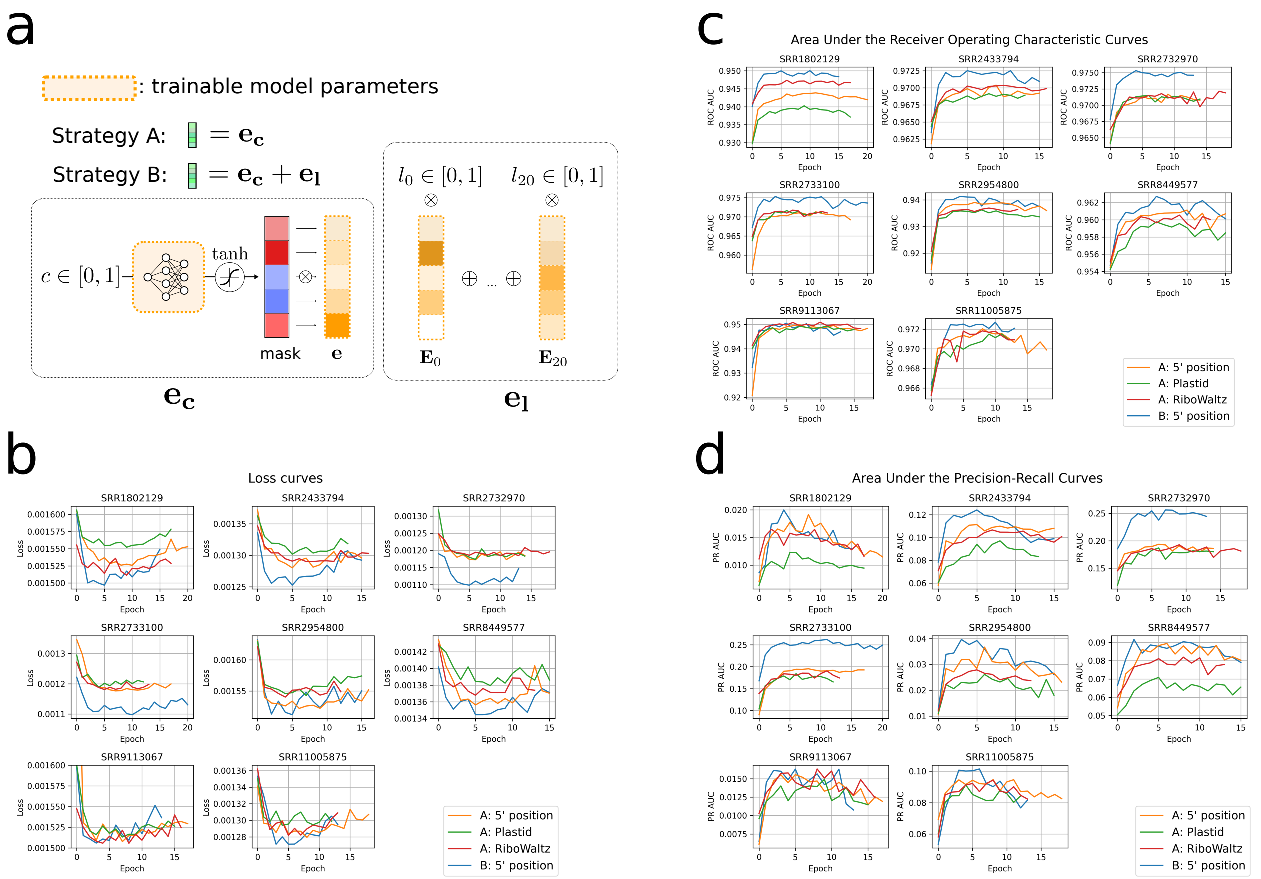

Supplementary Figure 6: **RiboTIE performances for different input token strategies and datasets.** **a**, Illustration of two different strategies for constructing the RiboTIE input vector. Strategy A: The input embedding ($\mathbf{e_c}$) for a given position is calculated using the total read count (normalized per transcript). This value is fed into a short feed-forward neural network. The tangens hyperbolicus transformation of the resulting vector functions as a mask for element-wise multiplication with a vector embedding **e** of equal size. Strategy B combines (i.e., sums) the input embedding from Strategy A with an embedding derived from the read lengths mapped to a given position. For a given input, read length embeddings are multiplied by the fractional representation of that read length at that position ($\mathbf{e_l}$). The latter is calculated by linear combination of the fractional representation of each read length ($\mathbf{l}$; for read lengths ranging from 20–40) with a dedicated vector embedding ($\mathbf{E}_0$–$\mathbf{E}_{20}$). Parameters optimized during the training process are displayed in orange. (**b**, **c**, **d**) Scores are calculated on the test set after selection of the model with the minimum validation loss. For each dataset and strategy, the cross-entropy loss, area under the receiver operating characteristic curve (ROC AUC), and area under the precision-recall curve (PR AUC) are given. Results indicate the relevance of read length information for the prediction of translation initiation sites using ribosome profiling data, especially for datasets featuring a higher read depth. All strategies are evaluated using the same model architecture and training/validation data. Strategy A has been evaluated for reads mapped by their 5'-end and reads offset based on read length information utilizing two different tools (Plastid, RiboWaltz).

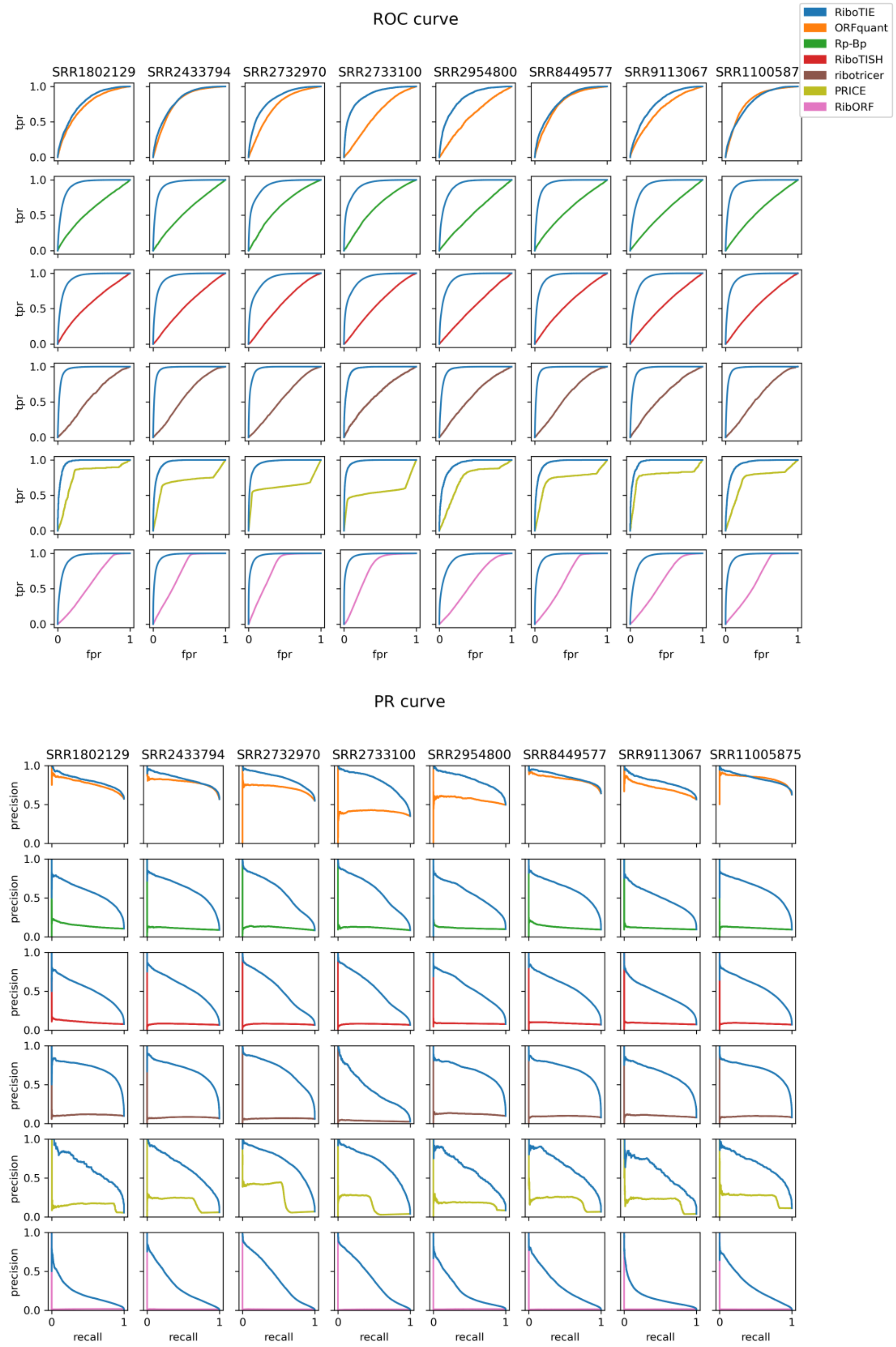

Supplementary Figure 7: **Receiver operating characteristics and Precision-recall curves for each of the evaluated datasets.** Performances are given for multiple datasets and tools. Unlike RiboTIE, other tools evaluate only a small selection of positions/ORFs on the transcriptome based on a variety of filters (e.g. start codon, ORF length, number of mapped reads...). Only these sites can be taken into account when calculating the score metric. Coding sequences annotated by Ensembl (GRCh38v110) function as the positive set with which the receiver operating characteristic curve (ROC) and precision-recall curve (PR) are calculated. Note that the number of positions and composition of positive and negative samples evaluated by each tool is unique and influences the score metric. Thus, performance scores are not comparable between tools.

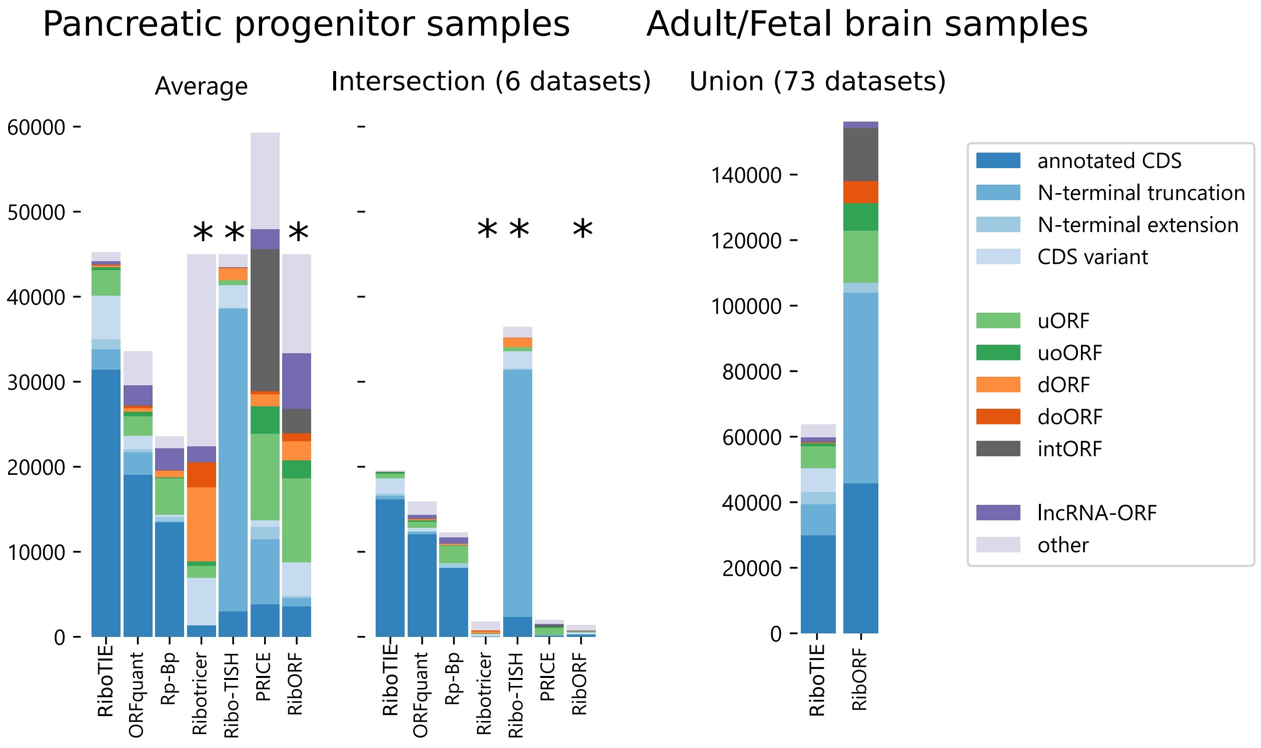

Supplementary Figure 8: **Stacked bar plot denoting the number of ORFs for each type within the positive set of various tools** on (**a**) the pancreatic progenitor cells (hESC) and (**b**) adult/fetal brain samples. Tools tagged with "*" (Ribotricer/Ribo-TISH/RibORF) give output predictions on all ORFs within their ORF libraries. As such, a positive set with an identical size to that of RiboTIE was selected for comparison by taking the top scoring predictions.

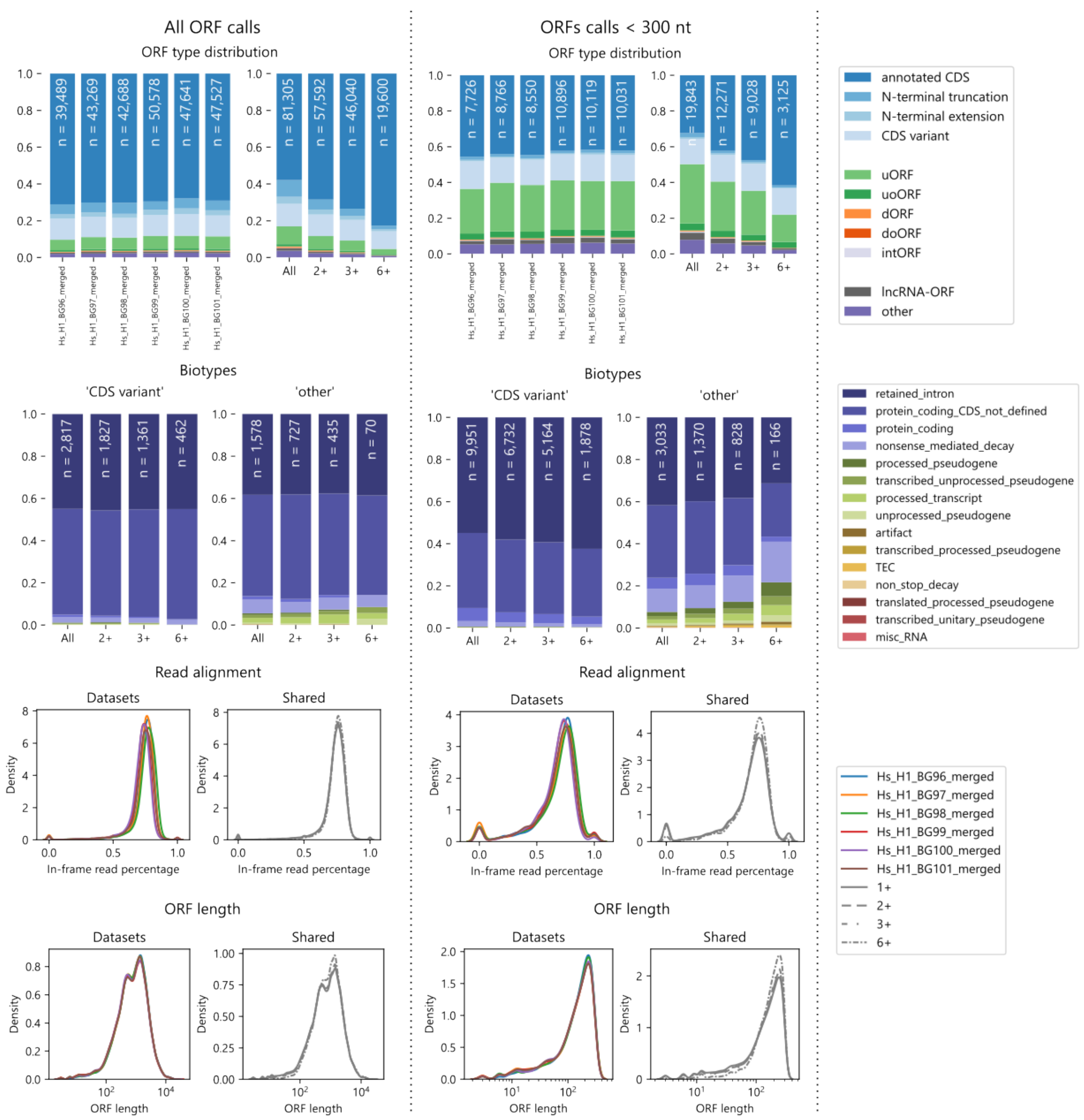

Supplementary Figure 9: **Properties of the called ORFs for the pancreatic progenitor cells.** Properties are given for all called ORFs (left), and those with a length smaller than 300 nucleotides (right). Shown are the ORF type distributions for the individual and merged sets. For the latter, the count reflects the number of unique ORFs present in any or at least 2/3/6 datasets. In-frame read percentages are with respect to the 5'-end of the reads, as no P-site calculation is performed by RiboTIE.

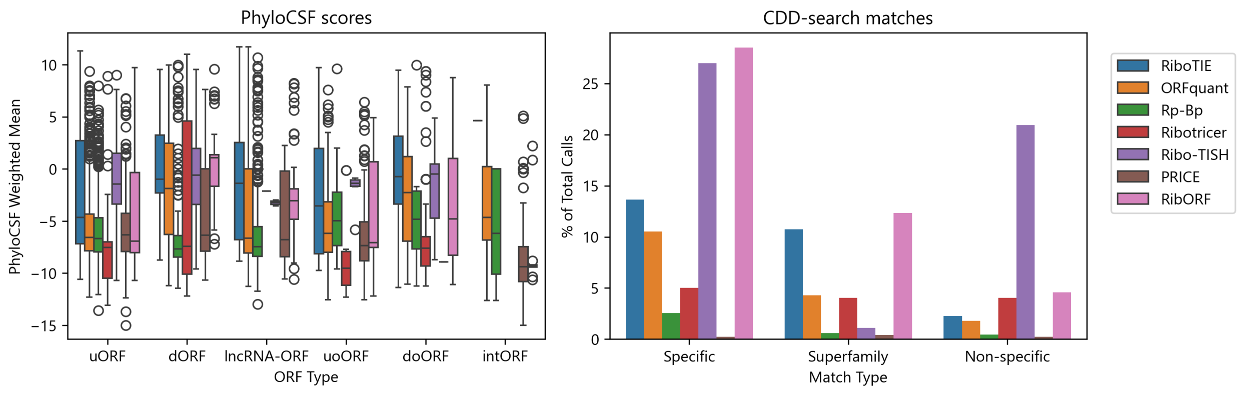

Supplementary Figure 10: **Evaluation of conservation patterns on ncORFs in pancreatic progenitor cells.** (left) PhyloCSF scores evaluate conservation of sequences across organisms. (right) Conserved Domain Database (CDD) searches evaluate the existence of known protein domains in amino acid sequences. It is important to notice that both PhyloCSF and CDD search matches are influenced by the approach with which tools filter ncORFs. For example, some tools might filter out likely false positive ncORF calls on transcript isoforms based on coordinates shared with annotated coding sequences, or based on transcript tags. Tools with less stringent filtering strategies are likely to score better. Only ncORFs that are called on all six replicate datasets are included. See Extended Data Table 3 for the list of called ncORFs.

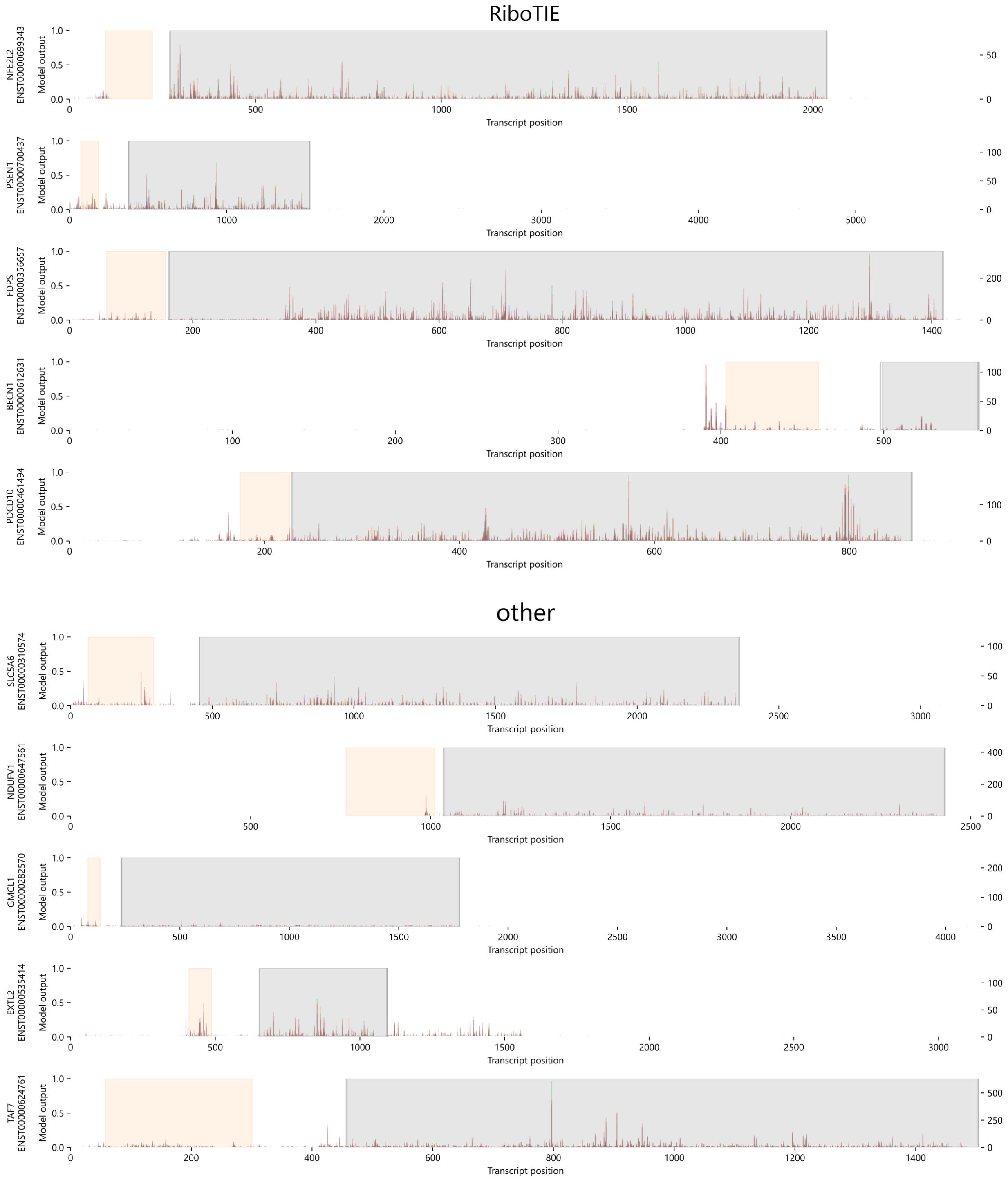

Supplementary Figure 11: **Examples of translated uORFs called exclusively by RiboTIE or other tools on pancreatic progenitor cells.** For RiboTIE (top), examples include called ORFs predicted on all six replicates while absent from any replicate by any other tool. For 'other' (bottom), examples include ORFs called by at least two other tools on all six replicates and absent from RiboTIE predictions on any of the six replicate datasets. Given are the ribosome read counts (y-axis) for the replicate pancreatic cells by positions on the transcript (x-axis). Read counts for the six replicate samples are represented by different colors (but generally overlapping). Areas covering both the predicted uORF (orange) and canonical sequence (gray) are given.

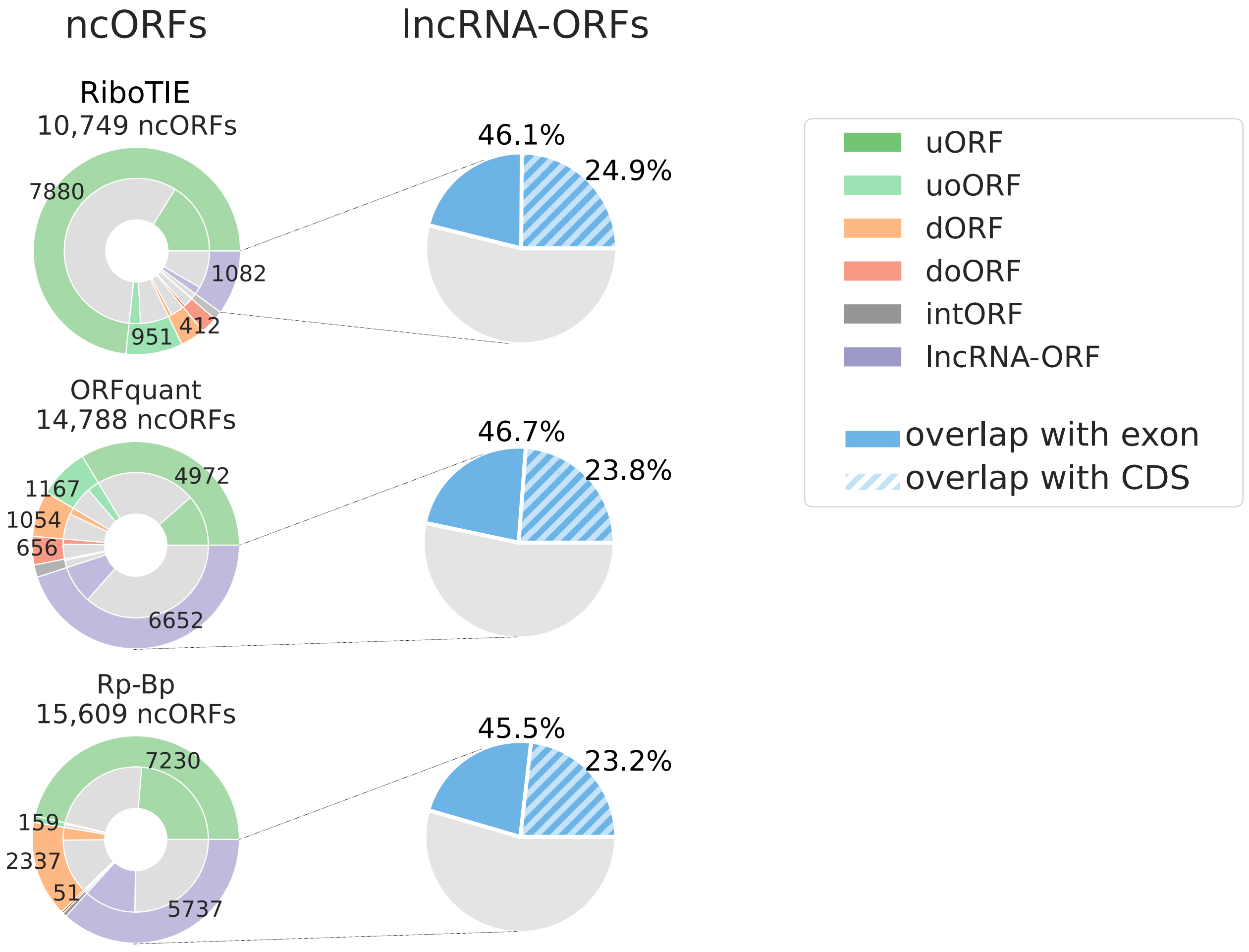

Supplementary Figure 12: **Characteristics of nominated lncRNA-ORFs by RiboTIE, ORFquant, and Rp-Bp on pancreatic progenitor cells.** Percentages reflect the number of lncRNA-ORFs that have any overlap with protein coding exons and CDSs based on the genomic coordinates of each of the regions.

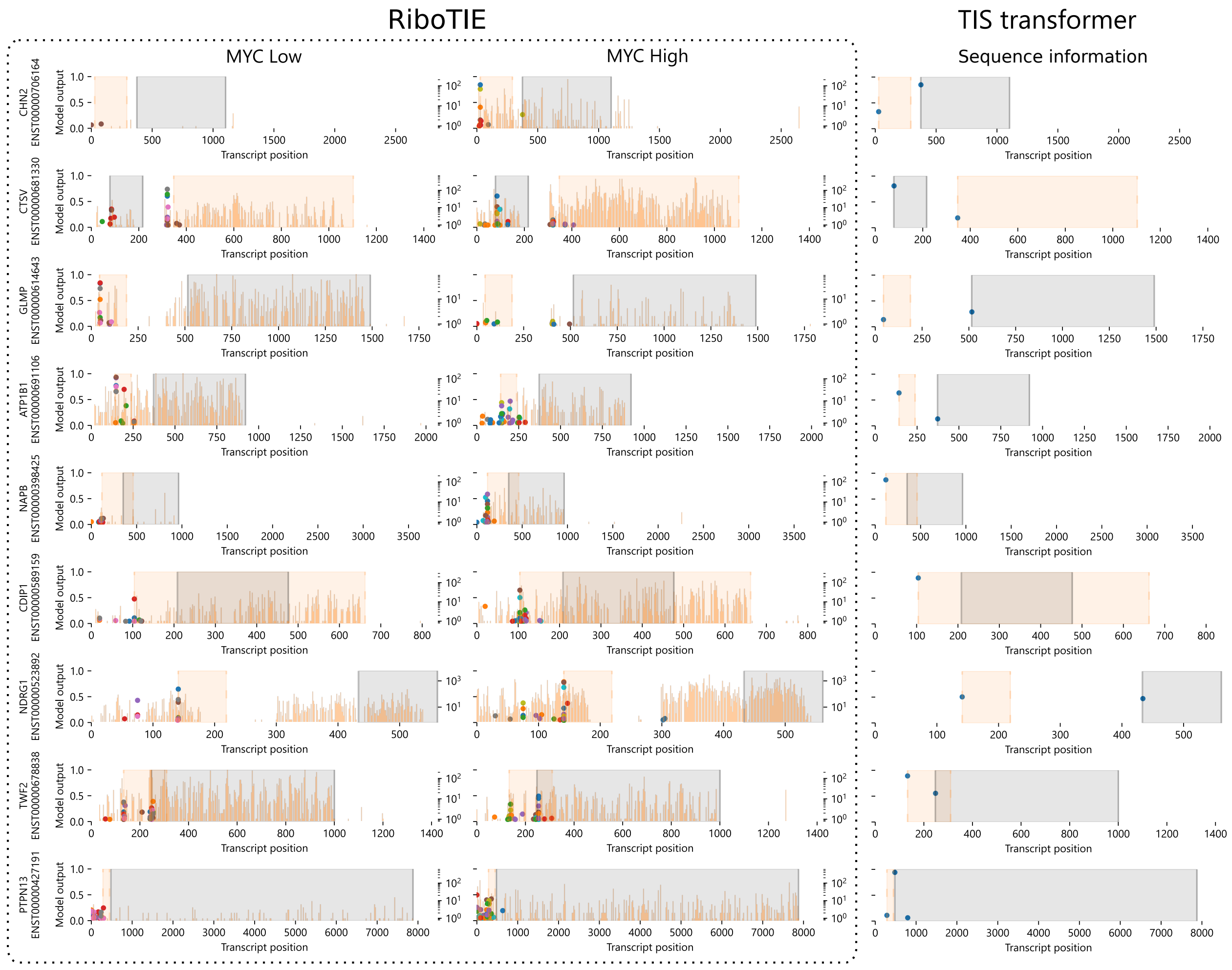

Supplementary Figure 13: **Example ncORFs with differential expression between medulloblastoma cell lines with high and low MYC expression.** Given are the model outputs (y-axis) for positions of the transcript (x-axis) where the output of the model (RiboTIE: left; TIS Transformer: right) is larger than 0.04. Both models detect TISs from which the resulting translated ORFs are derived. The model outputs for different samples are represented by different colors. The area of highly predicted ORFs is shown in grayss for annotated CDSs and orange otherwise. Ribo-Seq data, summed for all experiments in each group (low/high MYC), are displayed as orange bar plots (logarithmic scale, right y-axis).

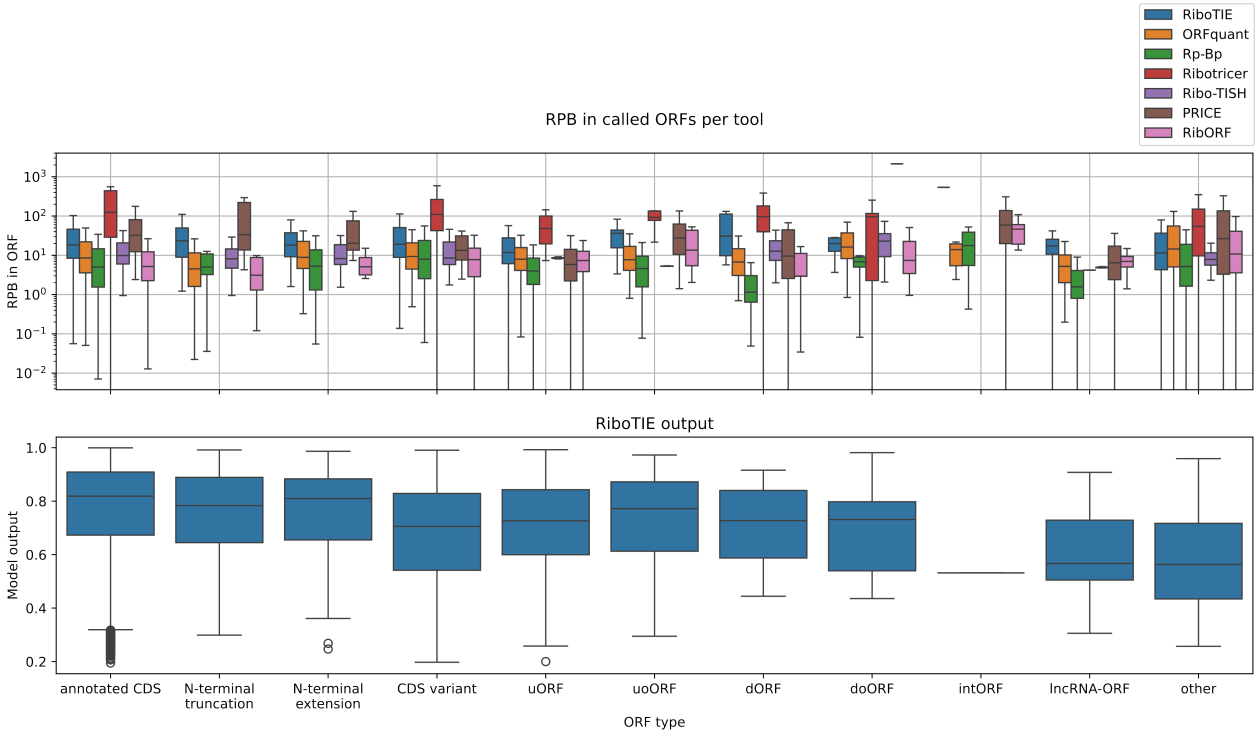

Supplementary Figure 14: **The correlation between ribosome reads and ORF types called on the pancreatic progenitor cells** (top) Reads per base (RPB) categorized by ORF type as called by each tool. (bottom) The model output distribution of RiboTIE for each of the ORF types. The data reveals lncRNA-ORFs to have lower RBPs as compared to the other subtypes, resulting in generally lower scores assigned by RiboTIE, and that tools with higher called lncRNA-ORFs detect these despite these low RPB values.

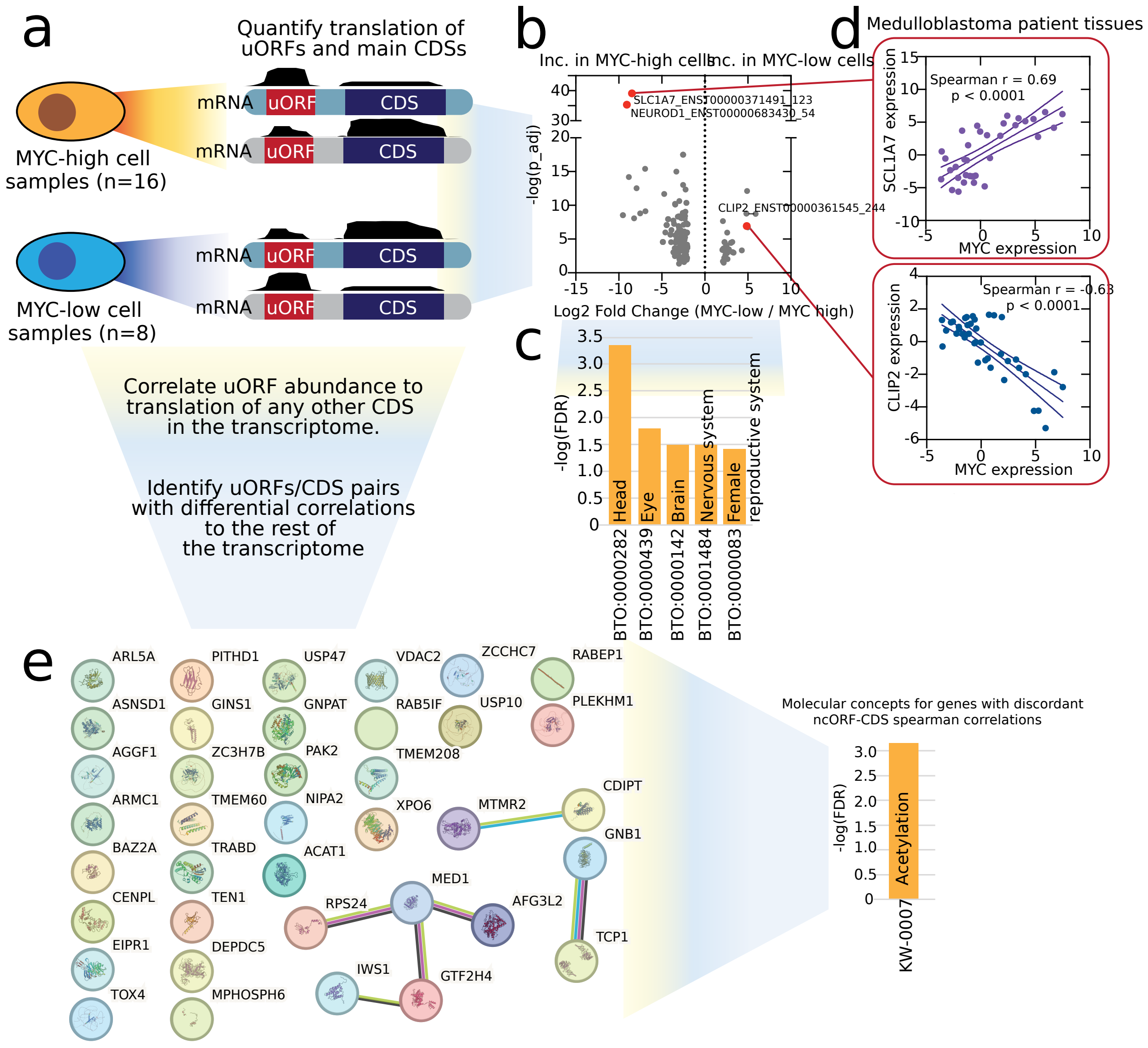

Supplementary Figure 15: **Biological associations of RiboTIE ncORFs in medulloblastoma.** **a**, A schematic showing the work-flow of comparing MYC-high and MYC-low medulloblastoma cell lines. **b**, A volcano plot showing 190 ncORFs with statistically-significant changes in translational abundance between MYC-high and MYC-low medulloblastoma cells. **c**, Network analysis of functional associations for the 109 genes that harbor the 190 ncORFs with differential translation, using String-db. **d**, Using medulloblastoma patient tissues (n=39) from the Clinical Proteome Tumor Analysis Consortium (CPTAC) (`10.1016/j.ccell.2018.08.004`), the correlation of mRNA expression for two differentially-regulated ncORFs nominated in medulloblastoma cell lines is shown. Spearman correlation coefficient and p value are indicated alongside the 95% confidence interval. **e**, Network analysis of 62 ncORFs residing in 39 Ensembl genes that are divergent genome-wide correlations between the uORF and the adjacent CDS (Extended Data Table 4). Network analysis is visualized using String-db.

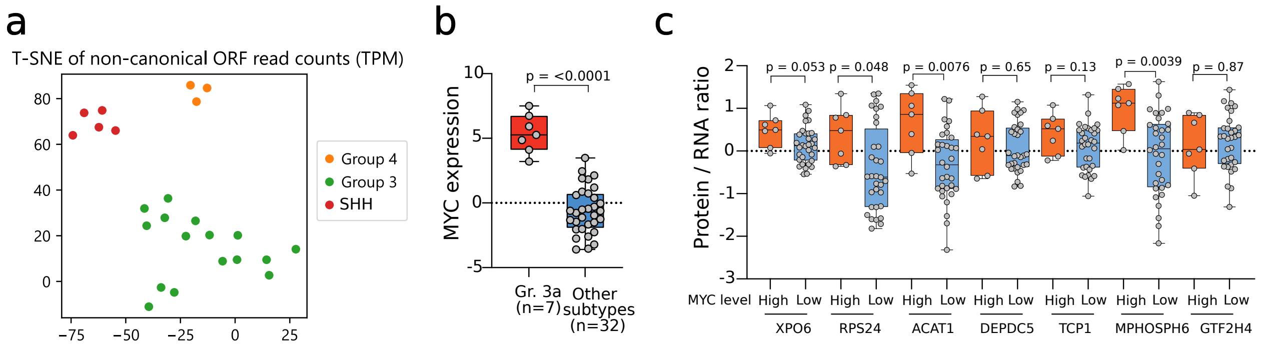

Supplementary Figure 16: **a**, Clustering of Medulloblastoma cell line samples on non-canonical ORFs as called by RiboTIE. Clustering is performed on the normalized number of mapped reads (Transcripts Per Million (TPM)) using both PCA (d=8) followed by T-SNE (Extended Data Table 4). Sonic Hedgehog activated (SHH) and groups 3 medulloblastoma samples have low MYC expression, where groups 3 has high MYC expression. **b**, Expression of MYC in previously defined MYC-high Group 3a medulloblastoma patients (n=7) and patients with other disease subtypes (n=32). P-value by a Mann Whitney U test. **c**, Protein-RNA dyssynchrony of 7 genes in 39 medulloblastoma patient tissues. Genes are selected as those that harbor ncORF-CDS pairs with inverse correlations between MYC-high and MYC-low cell lines (Extended Data Table 3). Protein-RNA dyssynchrony is stratified between patients with high MYC and low MYC expression (shown in panel b). P values are by a two-sided Mann Whitney U test.

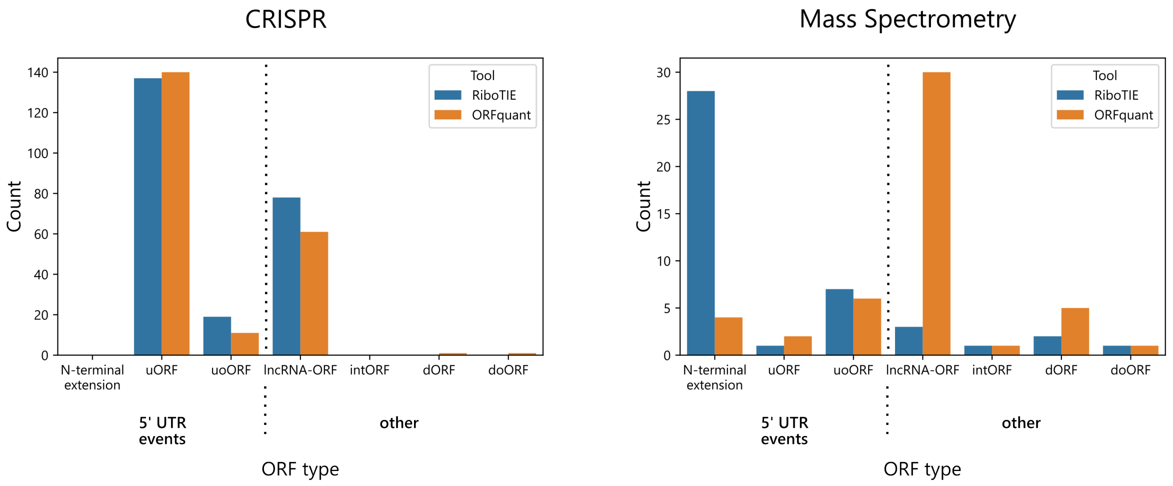

Supplementary Figure 17: **Matches between ORFs called by RiboTIE and ORFquant on the medulloblastoma cell line samples.** While CRISPR screen does not show any distinct differences between RiboTIE and ORFquant, the mass spectrometry (MS) data shows increased matches of N-terminal extensions for RiboTIE and lncRNA-ORFs for ORFquant. MS data was processed using FragPipe, where matches on called translated ORFs were filtered out if they were also matched to either Swiss-prot or annotated protein sequences (coding sequences) by Ensembl GRCh38.

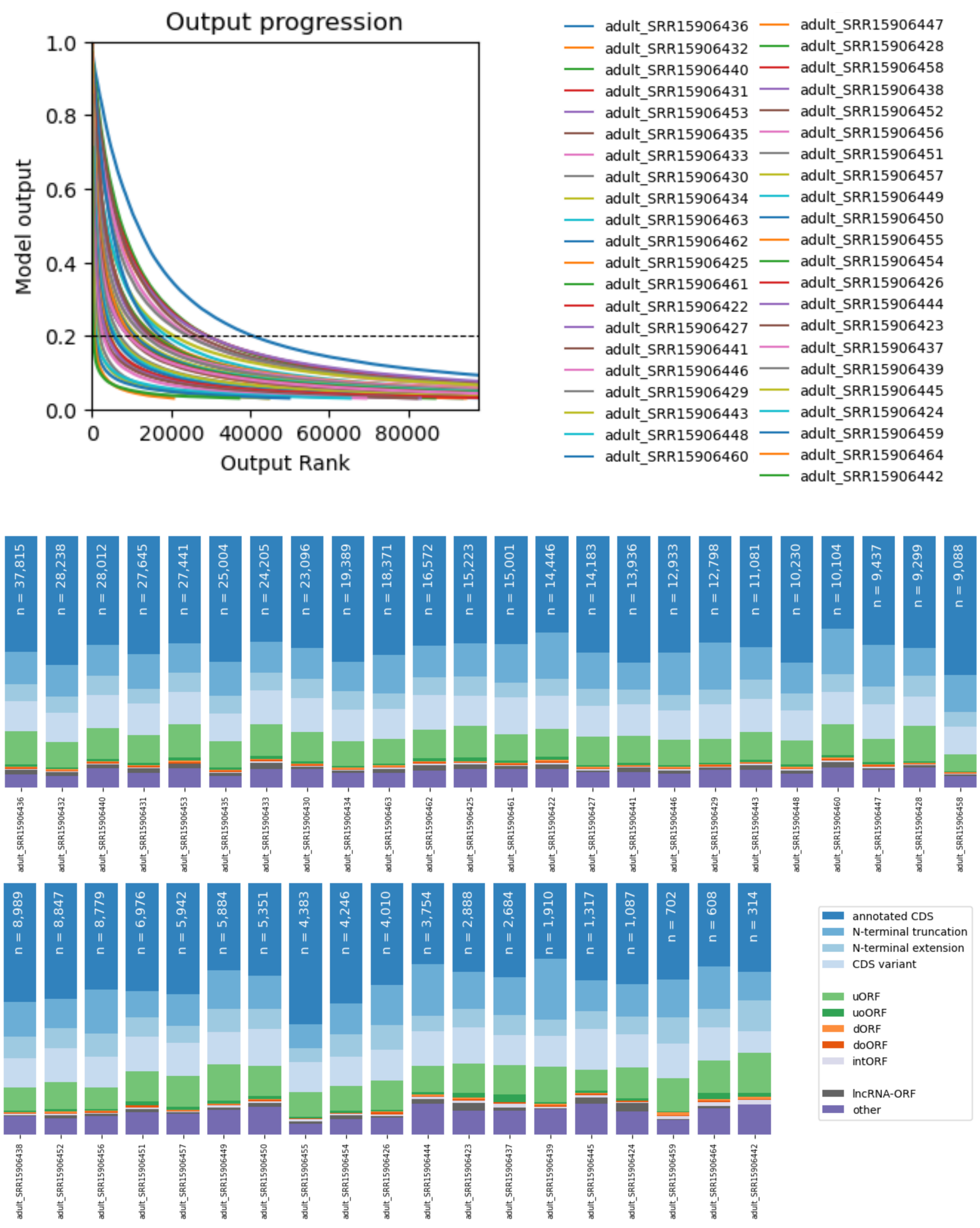

Supplementary Figure 18: **The distribution of ORF types returned by RiboTIE are stable for varying sequencing depths of the sample.** We find that RiboTIE returns stable accuracies independent of sequencing depth. Sequencing depth primarily affects the number of predictions crossing a certain threshold, rather than the composition of ORF types in the positive set. (top) For the adult brain samples generated by Duffy et al. (Extended Data Table 1), model output ranges are given for the top 100,000 predictions. (bottom) For predictions exceeding the threshold of 0.2, the fraction of each ORF type is given for each dataset. To correctly reflect the model behavior, the data was generated directly from the model output predictions, with no additional post-processing or filtering. The 'annotated CDS' ORF type is generally referred to as the positive set in the manuscript.

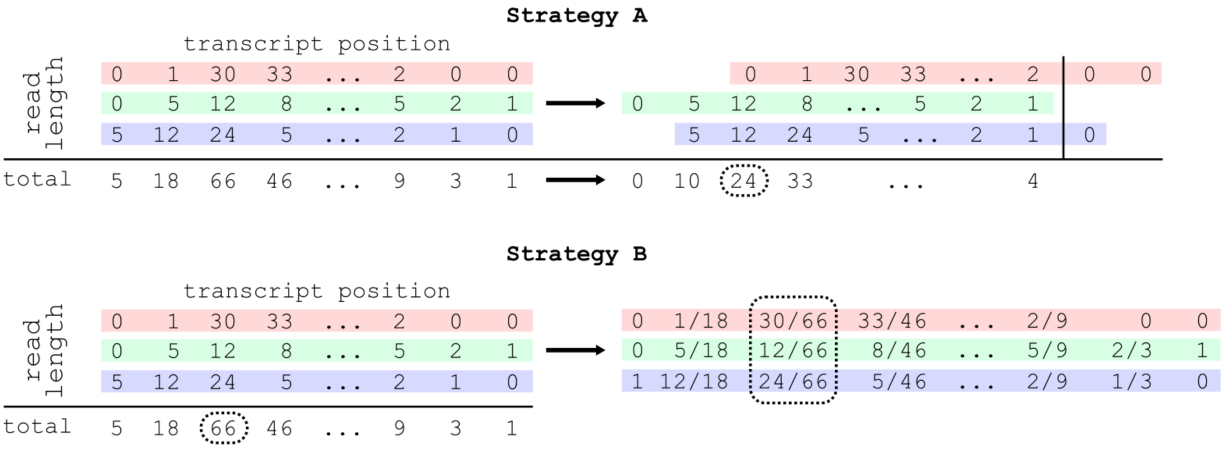

Supplementary Figure 19: **Illustration of the data applied for calculating the input vector representation.** For a given matrix containing reads mapped according to their 5'-end by transcript position and read length. Strategy A: reads are offset according to a fixed value for each read length. The total read count is applied for further processing. Strategy B: both the total read count and the fractional abundance of each read length is used to obtain an input vector representation. Input vector representations are calculated for each position (e.g. dotted square encapsulates data used for a single position). Note that in contrast to the illustration, data is generally sparse and ribosome profiling data is applied for 21 read lengths ($[20, 40]$).

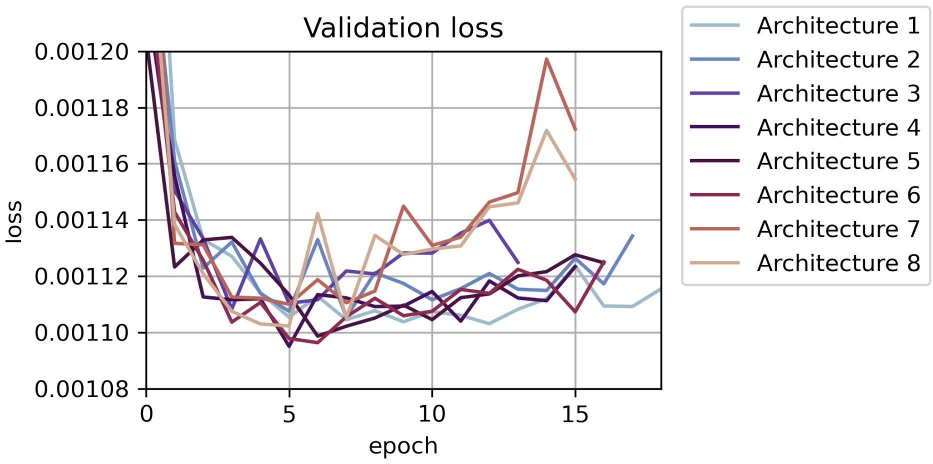

Supplementary Figure 20: **The loss curves of the model architectures trained for detecting TIS using ribosome profiling data.** The validation sets used are chromosomes 2 and 14. The hyperparameters for each model are given in Table 4. Architectures 1–2 converge slowly over several epochs without reaching a minimum within the evaluated time frame, indicating too few model weights. The higher number of model weights of architectures 7–8 result in clear overfitting from epoch 5 onward. Architecture 4 returns the lowest loss, and is selected for model benchmarking. While the minimum loss is similar for all architectures, the plot confirms our selection of a model architecture with a suitable number of parameters.

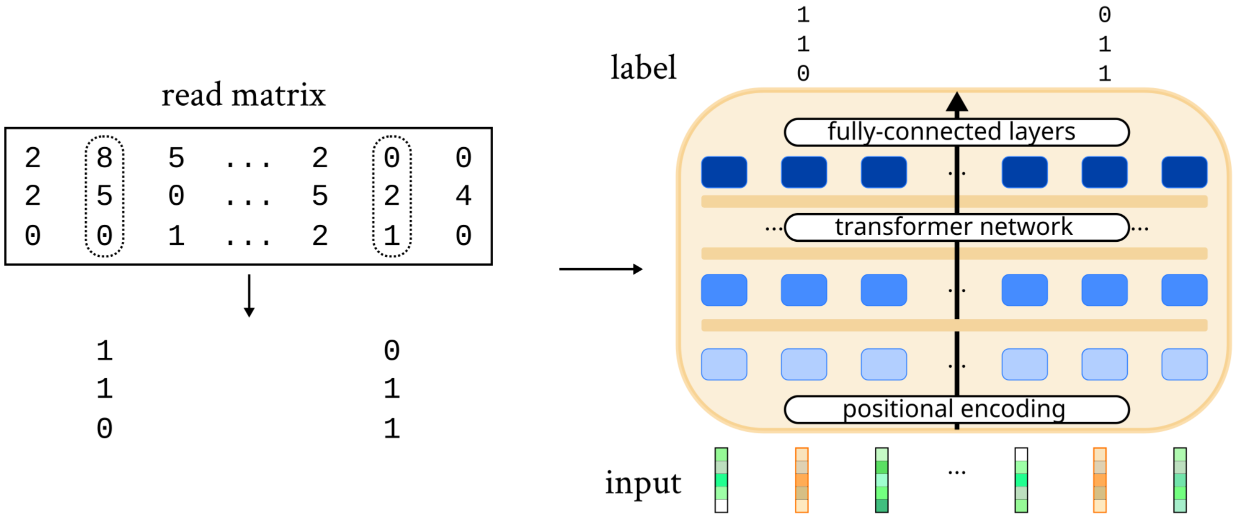

Supplementary Figure 21: **Self-supervised learning implementation for ribosome profiling data.** One of the pre-training approaches investigated in this paper. A model is trained to infer the presence of a mapped ribosome reads at a given position. The task constitutes a binary multi-label classification task. 15% of the input positions were randomly selected (dotted frame) and masked using a custom input embedding (orange input vectors). Positive labels are allocated to read lengths having more than one read mapped at a given position. Note that in contrast to the illustration, data is generally sparse and ribosome profiling data is applied for 21 read lengths ($[20, 40]$).

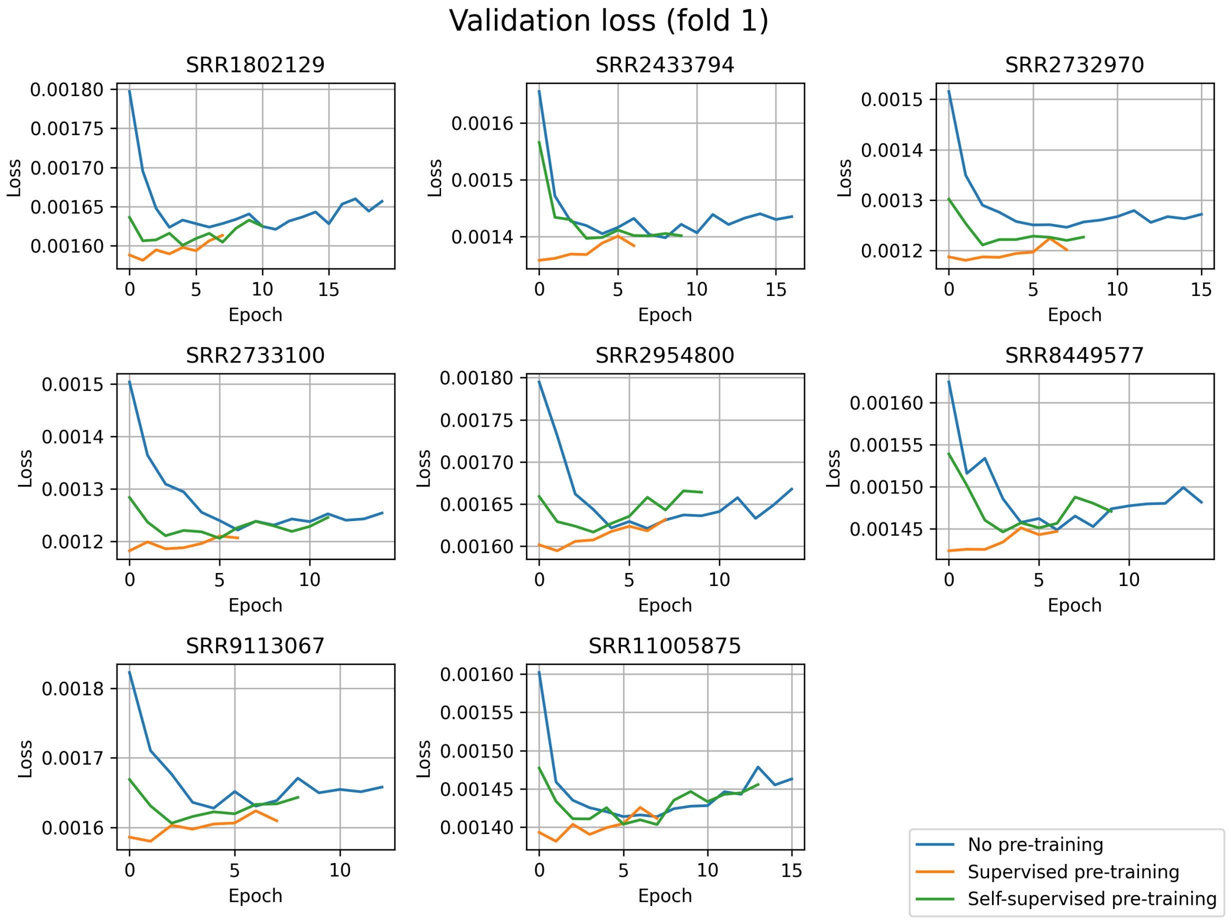

Supplementary Figure 22: **Validation cross-entropy loss of different training schemes of RiboTIE.** Eight datasets were evaluated following three approaches. This is achieved by training a model from scratch (no pre-training) or using a pre-trained model fit on a selection of eight separate datasets (see Extended Data Table 1). Pre-trained models include both those fit following a supervised learning objective (supervised pre-training) on identifying translation initiation sites, and those fit following a self-supervised learning objective, similar to those found in language processing (see Supplementary Figure 21). This figure shows the models trained on chromosomes 3, 5, 7, 11, 13, 15, 19, 21, and X with chromosomes 1, 9, and 17 used as validation set.

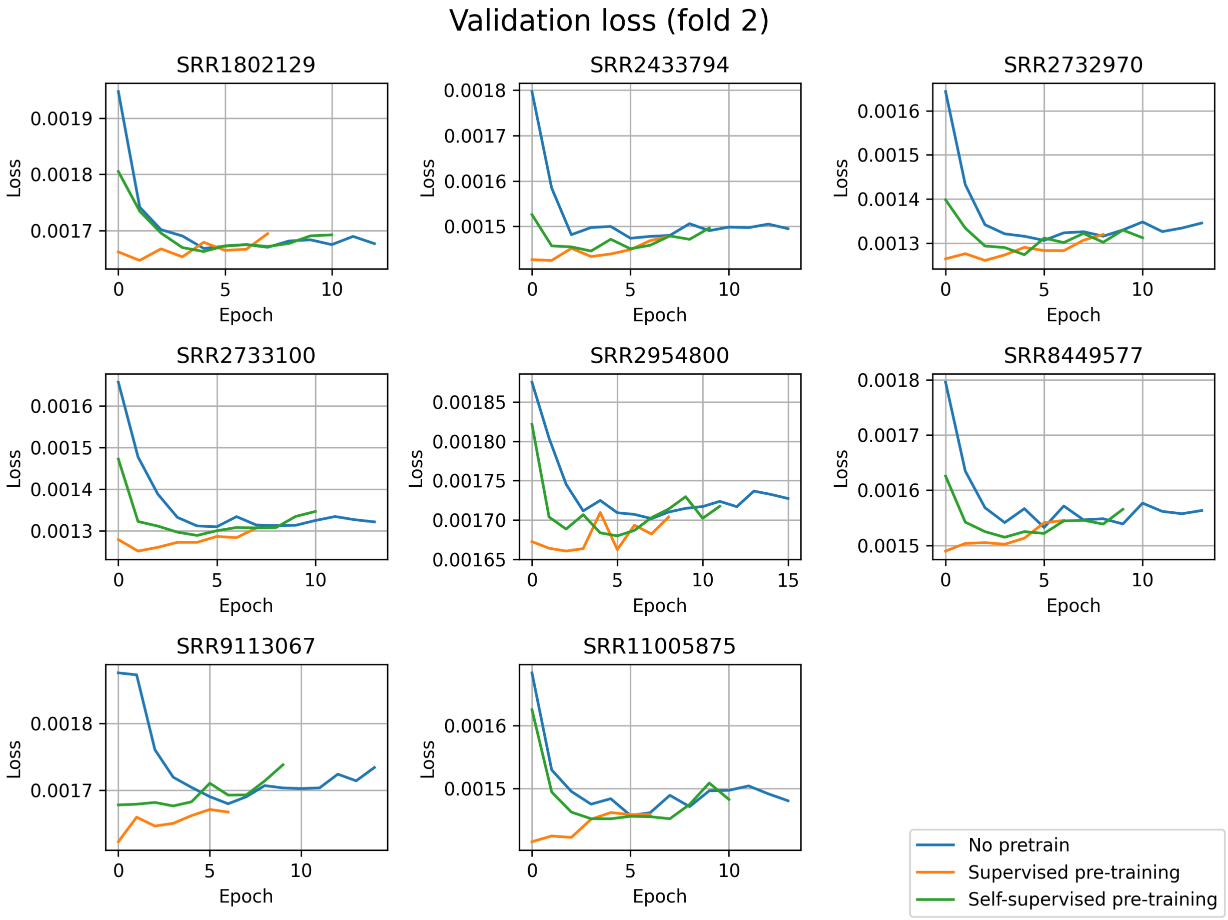

Supplementary Figure 23: **Validation cross-entropy loss of different training schemes of RiboTIE.** Eight datasets were evaluated following three approaches. This is achieved by training a model from scratch (no pre-training) or using a pre-trained model fit on a selection of eight separate datasets (see Extended Data Table 1). Pre-trained models include both those fit following a supervised learning objective (supervised pre-training) on identifying translation initiation sites, and those fit following a self-supervised learning objective, similar to those found in language processing (see Supplementary Figure 21). This figure shows the models trained on chromosomes 2, 6, 8, 10, 14, 16, 18, 22, and Y with chromosomes 4, 12, and 20 used as validation set.

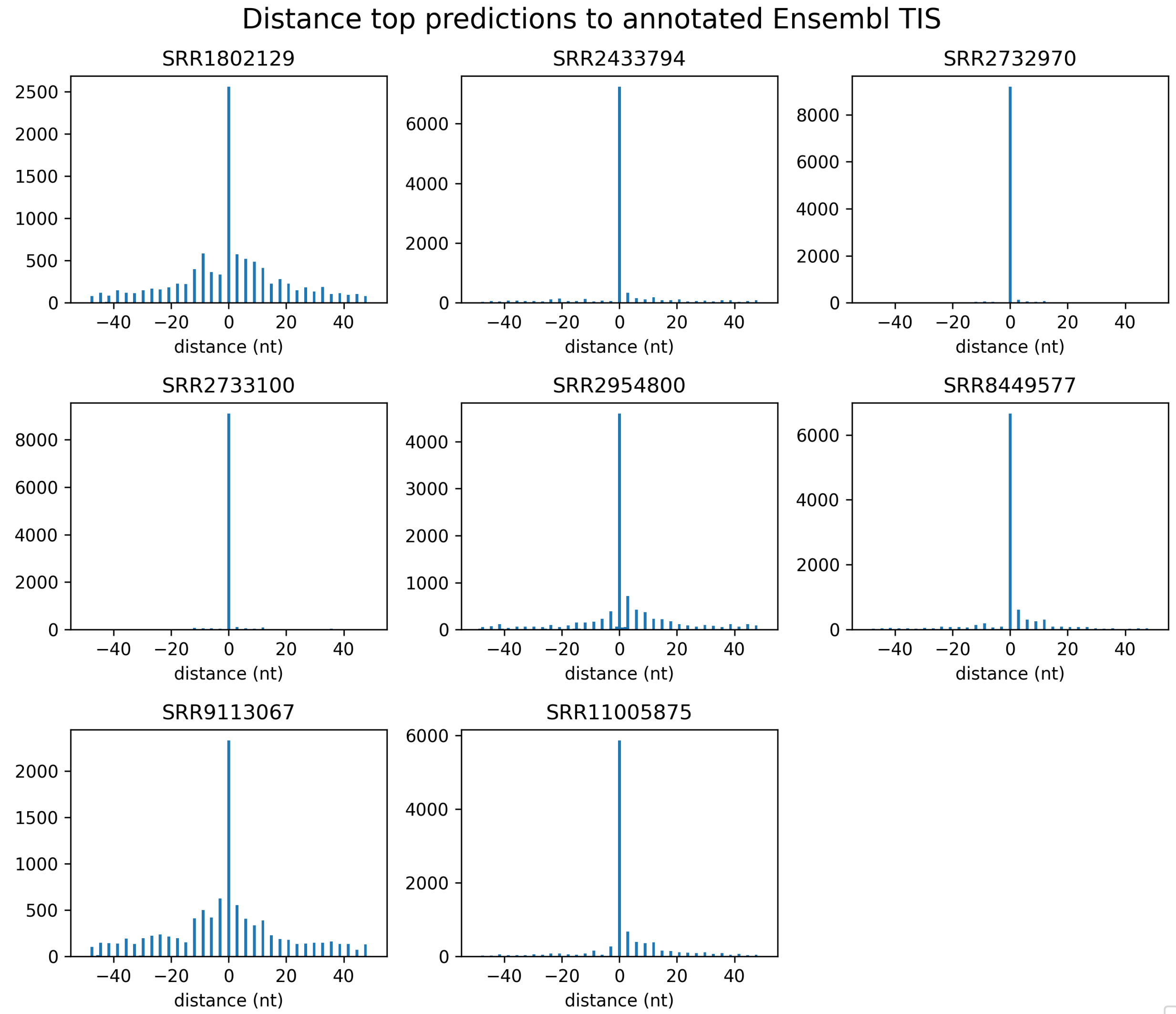

Supplementary Figure 24: **Accuracy of pinpointing translation initiation site (TIS) positions is correlated with read depth of ribosome profiling experiment.** Because RiboTIE does not pre-process candidate open reading frames or pass start codon information, pinpointing TISs can suffer for datasets with lower read depth. For each of the benchmark datasets, the histograms on distances between the a top-scoring RiboTIE prediction and an annotated Ensembl TIS is given.

Supplementary References

[1] Clauwaert, J., McVey, Z., Gupta, R. & Menschaert, G. TIS Transformer: Remapping the human proteome using deep learning. *NAR genomics and bioinformatics* **5**, lqad021 (2023).

[2] Choromanski, K. *et al.* Rethinking Attention with Performers. *arXiv:2009.14794 [cs, stat]* (2021). 2009.14794.

[3] Su, J. *et al.* RoFormer: Enhanced Transformer with Rotary Position Embedding (2022). 2104.09864.

[4] Choudhary, S., Li, W. & D. Smith, A. Accurate detection of short and long active ORFs using Ribo-seq data. *Bioinformatics* **36**, 2053–2059 (2020).

[5] Ahmed, N. *et al.* Identifying A- and P-site locations on ribosome-protected mRNA fragments using Integer Programming. *Scientific Reports* **9**, 6256 (2019).

[6] Lauria, F. *et al.* riboWaltz: Optimization of ribosome P-site positioning in ribosome profiling data. *PLOS Computational Biology* **14**, e1006169 (2018).

[7] Ji, Z. RibORF: Identifying Genome-Wide Translated Open Reading Frames Using Ribosome Profiling. *Current Protocols in Molecular Biology* **124**, e67 (2018).

[8] Xiao, Z. *et al.* De novo annotation and characterization of the translatome with ribosome profiling data. *Nucleic Acids Research* **46**, e61 (2018).

[9] Xu, Z. *et al.* Ribosome elongating footprints denoised by wavelet transform comprehensively characterize dynamic cellular translation events. *Nucleic Acids Research* **46**, e109 (2018).

[10] Fang, H. *et al.* Scikit-ribo Enables Accurate Estimation and Robust Modeling of Translation Dynamics at Codon Resolution. *Cell Systems* **6**, 180–191.e4 (2018).

[11] Malone, B. *et al.* Bayesian prediction of RNA translation from ribosome profiling. *Nucleic Acids Research* **45**, 2960–2972 (2017).

[12] Zhang, P. *et al.* Genome-wide identification and differential analysis of translational initiation. *Nature Communications* **8**, 1749 (2017).

[13] Dunn, J. G. & Weissman, J. S. Plastid: nucleotide-resolution analysis of next-generation sequencing and genomics data. *BMC Genomics* **17**, 958 (2016). URL http://dx.doi.org/10.1186/s12864-016-3278-x.

[14] Erhard, F. *et al.* Improved Ribo-seq enables identification of cryptic translation events. *Nature Methods* **15**, 363–366 (2018).

[15] Chun, S. Y., Rodriguez, C. M., Todd, P. K. & Mills, R. E. SPECtre: A spectral coherence-based classifier of actively translated transcripts from ribosome profiling sequence data. *BMC Bioinformatics* **17**, 482 (2016).

[16] Raj, A. *et al.* Thousands of novel translated open reading frames in humans inferred by ribosome footprint profiling. *eLife* **5**, e13328 (2016).

[17] Popa, A. *et al.* RiboProfiling: A Bioconductor package for standard Ribo-seq pipeline processing [version 1; peer review: 3 approved]. *F1000Research* **5** (2016).

[18] Calviello, L. *et al.* Detecting actively translated open reading frames in ribosome profiling data. *Nature Methods* **13**, 165–170 (2016).

[19] Fields, A. P. *et al.* A Regression-Based Analysis of Ribosome-Profiling Data Reveals a Conserved Complexity to Mammalian Translation. *Molecular Cell* **60**, 816–827 (2015).

[20] Crappé, J. *et al.* PROTEOFORMER: Deep proteome coverage through ribosome profiling and MS integration. *Nucleic Acids Research* **43**, e29 (2015).
