# Supplementary material for: Deep learning to decode sites of RNA translation in normal and cancerous tissues
Source: Nat Commun. 2025 Feb 2;16:1275. doi: 10.1038/s41467-025-56543-0 (PMC11788427; doi:10.1038/s41467-025-56543-0)
Supplement: Supplementary file 2 — Description of Additional Supplementary Files [file 41467_2025_56543_MOESM2_ESM.docx]

**Description of Additional Supplementary Files**

Supplementary Data Table 1: Datasets used in this study

Supplementary Data Table 2: Comparative performances of RiboTIE with different tools

Supplementary Data Table 3: ncORFs called in all 6 pancreatic progenitor cells, grouped for each tool.

Supplementary Data Table 4: Differentially expressed ncORFs between medulloblastoma cell lines with high and low MYC expression.

Supplementary Data Table 5: Gene network analysis results for the differentially expressed ncORFs called by RiboTIE

Supplementary Data Table 6: Mass spectrometry and CRISPR-screen results for ncORFs called in medulloblastoma data.
