# Supplementary material for: Deep learning to decode sites of RNA translation in normal and cancerous tissues
Source: Nat Commun. 2025 Feb 2;16:1275. doi: 10.1038/s41467-025-56543-0 (PMC11788427; doi:10.1038/s41467-025-56543-0)
Supplement: Supplementary file 9 — Reporting Summary [file 41467_2025_56543_MOESM9_ESM.pdf]

nature portfolio

Corresponding author(s): John Prensner

Last updated by author(s): Dec 17, 2024

Reporting Summary

Nature Portfolio wishes to improve the reproducibility of the work that we publish. This form provides structure for consistency and transparency in reporting. For further information on Nature Portfolio policies, see our Editorial Policies and the Editorial Policy Checklist.

Statistics

For all statistical analyses, confirm that the following items are present in the figure legend, table legend, main text, or Methods section.

n/a	Confirmed	
☐	☒	The exact sample size (*n*) for each experimental group/condition, given as a discrete number and unit of measurement
☐	☒	A statement on whether measurements were taken from distinct samples or whether the same sample was measured repeatedly
☐	☒	The statistical test(s) used AND whether they are one- or two-sided<br>*Only common tests should be described solely by name; describe more complex techniques in the Methods section.*
☐	☒	A description of all covariates tested
☐	☒	A description of any assumptions or corrections, such as tests of normality and adjustment for multiple comparisons
☐	☒	A full description of the statistical parameters including central tendency (e.g. means) or other basic estimates (e.g. regression coefficient) AND variation (e.g. standard deviation) or associated estimates of uncertainty (e.g. confidence intervals)
☐	☒	For null hypothesis testing, the test statistic (e.g. *F*, *t*, *r*) with confidence intervals, effect sizes, degrees of freedom and *P* value noted<br>*Give P values as exact values whenever suitable.*
☒	☐	For Bayesian analysis, information on the choice of priors and Markov chain Monte Carlo settings
☒	☐	For hierarchical and complex designs, identification of the appropriate level for tests and full reporting of outcomes
☐	☒	Estimates of effect sizes (e.g. Cohen's *d*, Pearson's *r*), indicating how they were calculated

Our web collection on statistics for biologists contains articles on many of the points above.

Software and code

Policy information about availability of computer code

Data collection	All software used in data collection were described in the Methods section of the paper. No specialized software or custom code was used for data collection.
Data analysis	Ribo-seq data was trimmed and mapped using STAR (v2.7.11a) and cutadapt (v4.4). Processing, machine learning and detection of translated ORFs was performed using the in-house RiboTIE package (transcript-transformer v0.3.2 for benchmark/hyperparameter selection/pretraining (DOI: 10.5281/zenodo.10689717, https://pypi.org/project/transcript-transformer/0.3.2/, https://github.com/TRISTAN-ORF/transcript_transformer/tree/0.3.2) , v0.5.6 (https://github.com/TRISTAN-ORF/transcript_transformer/tree/0.5.6, https://github.com/TRISTAN-ORF/transcript_transformer/tree/0.5.6) for subsequent reporting).<br>Ribo-seq offsets were determined using Plastid (v0.6.1) and RiboWaltz (v1.2.0), and benchmarking with other ORF callers using ORFquant (v1.02), Rp-Bp (v3.0.3), Ribo-TISH (v0.2.7), ribotricer (v1.3.3), price (GEDI package), and RibORF (v2.0).<br>Differential analysis was performed using PyDESeq2 (v. 0.4.4). Conserved Domain Database (CDD) search was performed on v3.21. Mass spectrometry analysis was performed using FragPipe v22.0.

For manuscripts utilizing custom algorithms or software that are central to the research but not yet described in published literature, software must be made available to editors and reviewers. We strongly encourage code deposition in a community repository (e.g. GitHub). See the Nature Portfolio guidelines for submitting code & software for further information.

Data

Policy information about availability of data

All manuscripts must include a data availability statement. This statement should provide the following information, where applicable:

- Accession codes, unique identifiers, or web links for publicly available datasets
- A description of any restrictions on data availability
- For clinical datasets or third party data, please ensure that the statement adheres to our policy

Data Availability:
The Medulloblastoma HHT Ribo-seq data generated in this study have been deposited in the Gene Expression Omnibus repository under accession code PRJNA1077309. The Mass spectrometry data generated in this study has been deposited in the ProteomeXchange under accession code PXD055854. Other existing data applied throughout the study includes those for the benchmark (SRR1802129, SRR2433794,SRR2732970, SRR2733100, SRR2954800, SRR8449577, SRR9113067, SRR11005875), model pre-training (SRR592960, SRR1562539, SRR1573939, SRR1610244, SRR1976443, SRR2536856, SRR2873532, SRR3575904), pancreatic progenitor cells (GSE144682), medulloblastoma cell lines (PRJNA957428) and tissue samples (phs003446), and fetal/adult brain samples (phs002489).

Research involving human participants, their data, or biological material

Policy information about studies with human participants or human data. See also policy information about sex, gender (identity/presentation), and sexual orientation and race, ethnicity and racism.

Reporting on sex and gender	NA
Reporting on race, ethnicity, or other socially relevant groupings	NA
Population characteristics	NA
Recruitment	NA
Ethics oversight	NA

Note that full information on the approval of the study protocol must also be provided in the manuscript.

Field-specific reporting

Please select the one below that is the best fit for your research. If you are not sure, read the appropriate sections before making your selection.

☒ Life sciences ☐ Behavioural & social sciences ☐ Ecological, evolutionary & environmental sciences

For a reference copy of the document with all sections, see nature.com/documents/nr-reporting-summary-flat.pdf

Life sciences study design

All studies must disclose on these points even when the disclosure is negative.

Sample size	For the RiboTIE design and benchmarking effort, a broad array of varying samples were selected to verify the robustness of the method. Performance evaluation is not performed in a traditional statistical sense, where machine learning model performances were evaluated with the idea that more data is better.<br>For evaluation of RiboTIE on the pancreatic stem cells, the data was selected in identical fashion to the previous use of the data for evaluating multiple ORF callers (10.1016/j.mcpro.2023.100631)<br>For the medulloblastoma data, samples were selected in line with in-house availability<br>For the mass spectrometry data, 3 samples per group (MYC-low/MYC-high) were selected adhering to the recommended minimum for unveiling differences in distributions statistically. In addition, three technical replicates (see replication) were taken.
Data exclusions	No data was excluded from the analyses.
Replication	For the benchmarking of RiboTIE with previous tools, a variety of datasets were selected to confirm RiboTIE consistently outperforms previous methods.<br>For the evaluation of RiboTIE on the pancreatic stem cells, ribosome profiling was merged for the five technical replicates featured for each biological replicate, in line with previous publications (10.1016/j.mcpro.2023.100631). Performance of the tool was seen to be highly reproducible.<br>For the mass spectrometry data, we took three technical replicates for each sample and averaged these. The three replicates showed high reproducibility amongst all groups.
Randomization	No randomization was applied on the data.

Blinding | Blinding is not relevant in this study design as no experiments were influenced by factors such as subjective evaluation and strong expectations which could bias the approach of the experiments performed.

Reporting for specific materials, systems and methods

We require information from authors about some types of materials, experimental systems and methods used in many studies. Here, indicate whether each material, system or method listed is relevant to your study. If you are not sure if a list item applies to your research, read the appropriate section before selecting a response.

Materials & experimental systems

n/a	Involved in the study
☒	☐ Antibodies
☐	☒ Eukaryotic cell lines
☒	☐ Palaeontology and archaeology
☒	☐ Animals and other organisms
☐	☒ Clinical data
☒	☐ Dual use research of concern
☒	☐ Plants

Methods

n/a	Involved in the study
☒	☐ ChIP-seq
☒	☐ Flow cytometry
☒	☐ MRI-based neuroimaging

Eukaryotic cell lines

Policy information about cell lines and Sex and Gender in Research

Cell line source(s)

Cell lines utilized by this study are all derived from medulloblastoma:
Sample Name Source Identifier(s) Biosample
DAOY ATCC Cat#HTB-186; RRID: CVCL_1167 SAMN34359890
CHLA-01-MEDR ATCC Cat#CRL-3034; RRID: CVCL_N534 SAMN34250959
D425 Bandopadhayay lab RRID: CVCL_1275 SAMN34359879
D458 Bandopadhayay lab RRID: CVCL_1161 SAMN34359878
D341 ATCC Cat#HTB-187; RRID: CVCL_0018 SAMN34359880
D384 CCLE RRID: CVCL_1157 SAMN34359882
MB002 (SUMB002) Bandopadhayay lab RRID: CVCL_VU79 SAMN34359885
R256 CCLE RRID: CVCL_DG09 SAMN34359887
R262 CCLE RRID: CVCL_VU83 SAMN34359886
CHLA-259 Children's Oncology Group Cat#CHLA-259; RRID: CVCL_M148 SAMN34359891
Med2112 Brain Tumor Resource Lab https://www.seattlechildrens.org/research/centers-programs/childhood-cancer/our-labs/jim-olson-lab/btrl/ SAMN34359884
Med411 Brain Tumor Resource Lab https://www.seattlechildrens.org/research/centers-programs/childhood-cancer/our-labs/jim-olson-lab/btrl/ SAMN34359883
D283Med ATCC Cat#HTB-185; RRID: CVCL_1155 SAMN34359881
ONS76 CCLE RRID: CVCL_1624 SAMN34359888
UW228 CCLE RRID: CVCL_8585 SAMN34359889

Authentication

Cell line authentication is maintained by STR profiling through LabCorp every 3-6 months

Mycoplasma contamination

Cell lines were tested for mycoplasma upon receipt and were negative.

Commonly misidentified lines (See ICLAC register)

No commonly mis-identified cell lines were used in this study.

Clinical data

Policy information about clinical studies

All manuscripts should comply with the ICMJE guidelines for publication of clinical research and a completed CONSORT checklist must be included with all submissions.

Clinical trial registration

Provide the trial registration number from ClinicalTrials.gov or an equivalent agency.

Study protocol

Note where the full trial protocol can be accessed OR if not available, explain why.

Data collection

Describe the settings and locales of data collection, noting the time periods of recruitment and data collection.

Outcomes

Describe how you pre-defined primary and secondary outcome measures and how you assessed these measures.

Plants

Seed stocks	*Report on the source of all seed stocks or other plant material used. If applicable, state the seed stock centre and catalogue number. If plant specimens were collected from the field, describe the collection location, date and sampling procedures.*
Novel plant genotypes	*Describe the methods by which all novel plant genotypes were produced. This includes those generated by transgenic approaches, gene editing, chemical/radiation-based mutagenesis and hybridization. For transgenic lines, describe the transformation method, the number of independent lines analyzed and the generation upon which experiments were performed. For gene-edited lines, describe the editor used, the endogenous sequence targeted for editing, the targeting guide RNA sequence (if applicable) and how the editor was applied.*
Authentication	*Describe any authentication procedures for each seed stock used or novel genotype generated. Describe any experiments used to assess the effect of a mutation and, where applicable, how potential secondary effects (e.g. second site T-DNA insertions, mosiacism, off-target gene editing) were examined.*
